# Active Starch Films Incorporated with Citrus Essential Oils: Properties, Bioactivity, and Biodegradability

**DOI:** 10.3390/polym18141794

**Published:** 2026-07-22

**Authors:** Jasamim Moreira Lemos, José Elias Machado Lopes, José Hilton Gomes Rangel, Sebastião Pereira Protázio, Gricirene Sousa Correia, Samuel Filgueiras Rodrigues, Walter José Martinez Burgos, Paula Beatricy Weba Moreira, Kiany Sirley Brandão Cavalcante, Josilene Lima Serra Pereira

**Affiliations:** 1 Graduate Program in Materials Science and Technology (PPGCTM) and Graduate Program in Chemistry (PPGQ), Federal Institute of Education, Science and Technology of Maranhão (IFMA), São Luís 65030-005, MA, Brazil; lemosjasamim@gmail.com (J.M.L.); sebastiaoprotazio@gmail.com (S.P.P.); gricirene.correia@ifma.edu.br (G.S.C.); samuel.filgueiras@ifma.edu.br (S.F.R.); paula.moreira@ifma.edu.br (P.B.W.M.); 2 Postgraduate Program in Chemistry, Federal Institute of Education, Science and Technology of Maranhão (IFMA), São Luís 65030-005, MA, Brazil; jose.machado@ifma.edu.br (J.E.M.L.); hiltonrangel@ifma.edu.br (J.H.G.R.); kiany@ifma.edu.br (K.S.B.C.); 3 Faculty of Engineering, Universidade Andres Bello, Av. Antonio Varas, Santiago 880, Chile; ing.wjmartinez@gmail.com; 4 Postgraduate Program in Materials Engineering and Postgraduate Program in Chemistry, Federal Institute of Education, Science and Technology of Maranhão (IFMA), São Luís 65030-005, MA, Brazil

**Keywords:** starch-based films, biodegradable packaging, citrus essential oils, active food packaging, antimicrobial properties, soil biodegradation

## Abstract

The demand for sustainable food preservation has driven the development of biodegradable alternatives to conventional, petroleum-based plastics. This study developed and evaluated bioactive starch-based films incorporated with citrus peel essential oils (lemon, orange, and tangerine) at concentrations ranging from 0.5% to 2% (*w*/*w*). The physical, chemical, mechanical, and antimicrobial properties of all film formulations containing 0.5–2% (*w*/*w*) essential oils were evaluated. Based on the experimental design, only the selected formulations containing 1 and 2% essential oils were subjected to structural and thermal characterization (XRD, SEM, FTIR, and TGA/DTG), transparency measurements, soil biodegradation, and phytotoxicity assays. FTIR spectroscopy revealed that oil incorporation did not alter the characteristic chemical bands of starch, indicating predominantly physical interactions. The essential oils modulated the physical and mechanical performance of the films. The films containing lemon and tangerine essential oils exhibited superior antimicrobial activity against foodborne pathogens. Furthermore, soil biodegradation was concentration-dependent, with mass loss exceeding 50% within 15 days, while phytotoxicity tests confirmed the environmental safety of the degraded residues. These findings demonstrate that the developed citrus-infused starch films hold great promise as active biodegradable packaging to extend the shelf life of bakery products and mitigate plastic waste.

## 1. Introduction

The environmental impact of the growing use of flexible plastic packaging, primarily used for short-shelf-life foods, has driven research toward sustainability initiatives, such as bioplastics and recyclable materials. Conventional petroleum-based plastics pose severe environmental threats, including ecosystem pollution, waste accumulation and microplastic contamination [1]. In this context, starches from various commercial sources, such as corn, potatoes, rice, wheat, and cassava, have been widely explored for developing biodegradable films and coatings because of their renewable nature, low cost, and availability [2]. However, despite their good optical properties, flexibility, and biodegradability, neat starch films have limitations, including poor mechanical strength and high water vapor permeability. To overcome these drawbacks, the incorporation of hydrophobic compounds and functional additives has emerged as a key strategy [3].

Recent trends in polymer science focus on developing active packaging by incorporating bioactive compounds into natural polymeric matrices [4]. These active films interact dynamically with the enclosed environment by releasing functional components or neutralizing deteriorative factors, thereby maintaining food safety [5]. A wide range of natural bioactive agents, including plant extracts, vitamins, and essential oils, have been successfully incorporated into biopolymers [6]. Concurrently, essential oils extracted from citrus fruit peels (such as lemon, orange, and tangerine) represent a valuable source of bioactive phytocompounds with well-documented antimicrobial and antioxidant activities [7]. Furthermore, utilizing these oils aligns with circular economy principles, as they are upcycled from agro-industrial waste generated by the juice industry, where peels and seeds account for tons of discarded biomass annually [8].

The application of citrus essential oils in biodegradable packaging has expanded considerably during the last few years [9,10]. Odjo et al. [11] developed chitosan-based antimicrobial films containing encapsulated lemon essential oil and demonstrated improvements in antioxidant activity, UV barrier properties and antifungal performance, emphasizing that encapsulation reduced the rapid volatilization of the oil while maintaining its biological activity. Likewise, Doğan et al. [12] incorporated lemon peel essential oil into gelatin micro–nanofibers and reported pronounced inhibition of *Staphylococcus aureus* and *Escherichia coli*, successfully extending cheese shelf life, although the antimicrobial effect was more pronounced against Gram-positive bacteria. Akachat et al. [13] produced pectin films containing encapsulated essential lemon oil and observed improvements in antioxidant capacity, antimicrobial activity, flexibility and biodegradability. In starch-based systems, Shamsabadi [14] reinforced starch films with cellulose nanofibers and lemon essential oil, reporting enhanced mechanical properties together with antibacterial activity. More recently, Zhang et al. [15] demonstrated that citrus-derived fibers significantly improved the barrier properties and controlled release behavior of starch composites, highlighting the potential of citrus-processing by-products for sustainable packaging applications.

Although the individual benefits of certain citrus oils are documented, comparative assessments conducted under identical formulation and processing conditions remain limited. Specifically, how the distinct volatile profiles of lemon, orange, and tangerine essential oils differentially modulate the internal macromolecular arrangement and subsequent transport properties of starch matrices remain poorly understood. Moreover, while previous studies have mainly focused on mechanical, barrier, and antimicrobial properties, few have concurrently established complete lifecycle analysis establishing both the polymer biodegradation kinetics and the ecotoxicological safety of the resulting postdegradation residues.

Therefore, this study aimed to develop and characterize bioactive starch-based films incorporated with citrus peel essential oils at different concentrations (1% and 2% *w*/*w*), focusing on the transfer of bioactivity to the polymeric matrix. The results demonstrated that while the physical integrity and characteristic chemical bands of starch were preserved, the films containing lemon and tangerine oils exhibited superior antimicrobial activity against target pathogens. Additionally, the films maintained high soil biodegradability with eco-friendly residues, demonstrating that the successful transfer of citrus bioactivity yields promising, environmentally safe functional materials.

## 2. Materials and Methods

### 2.1. Extraction, Yield, and Density of Essential Oils

Citrus peels from lemon (*Citrus latifolia* Tanaka), orange (*Citrus sinensis*), and tangerine (*Citrus reticulata*) were purchased from a local market in São Luís, MA, Brazil. The fruits were sanitized using a sodium hypochlorite solution (3:10 *v*/*v*). Fresh peels were separated into 300 g portions, vacuum-packed (GSVAC, model GS420, Santo André, Brazil) in polyethylene bags, and stored under refrigeration until extraction. The essential oils (EOs) were extracted by hydrodistillation using a Clevenger-type apparatus (Fireglass, Florianopolis, Brazil). A 4 L flat-bottom flask containing the crushed peels and filtered water in a 1:10 (*w*/*v*) ratio was heated to boiling at 100 °C for 4 h. The obtained EOs were dried over anhydrous sodium sulfate (Na_2_SO_4_; P.A.–ACS, Dinâmica Química Contemporânea Ltda., Indaiatuba, SP, Brazil) and stored in amber vials under refrigeration. The wet-base extraction yield was calculated using Equation (1) [16]:

Yield (%) = V_0_/B_m_ × 100 (1)

where V_0_ is the volume of essential oil obtained (mL) and B_m_ represents the plant biomass (g). The absolute density (g/mL) at 20 °C was determined using a 1 mL pycnometer. The relative density (d) was calculated according to Equation (2):

d = [(m_2_ − m_1_)/(m_3_ − m_1_)] × 100 (2)

where m_1_ is the mass of the empty pycnometer (g), m_2_ is the mass of the pycnometer filled with EO (g), and m_3_ is the mass of the pycnometer filled with distilled water (g).

### 2.2. Chemical Composition by Gas Chromatography–Mass Spectrometry (GC-MS)

Chromatographic analyses were performed at the Analytical Center of the Institute of Chemistry, University of São Paulo. The chemical profile of the EOs was determined using a GC-MS system (Shimadzu, model GCMS-QP2020, Kyoto, Japan) equipped with a split injector (1:5 ratio) maintained at 280 °C. Helium was used as the carrier gas at a constant linear velocity of 48.9 cm/s (flow rate of 1.80 mL/min and operating pressure of 16.2 psi). The initial oven temperature was set to 60 °C. Volatile compounds were identified by comparing their mass spectra with the NIST105, NIST21, and WILEY139 databases.

### 2.3. Antibacterial Activity of Essential Oils

Antibacterial activity was evaluated using the disk diffusion method [17] against *Escherichia coli* (ATCC 25922) and *Staphylococcus aureus* (ATCC 29213). Bacterial suspensions were prepared in Brain Heart Infusion (BHI) broth and adjusted to the McFarland scale (approx. 10^7^ CFU/mL). The inocula were spread onto Müller–Hinton agar plates, and sterile filter paper disks impregnated with the respective EOs were applied. The plates were incubated at 37 °C for 24 h, after which the inhibition zones were measured using a millimeter ruler. Commercial gentamicin disks (20 µg/g) served as the control, classified as Resistant (R ≤ 12 mm), Intermediate (I = 13–14 mm), or Susceptible (S ≥ 15 mm) [18]. The minimum inhibitory concentration (MIC) was determined by the broth microdilution method in 96-well plates [19]. BHI broth was supplemented with an initial EO concentration of 16%, followed by twofold serial dilutions (1:1 *v*/*v*) to yield concentrations ranging from 16% to 0.125%. Aliquots of 0.1 mL of the standardized bacterial suspension were inoculated into each well. After incubation at 37 °C for 24 h, the MIC was recorded as the lowest concentration capable of completely inhibiting visible bacterial growth.

### 2.4. Antifungal Activity of Essential Oils

The antifungal effect was evaluated against *Aspergillus niger*. Fungal suspensions were prepared in Yeast Peptone Glucose (YPG) broth and inoculated onto Potato Dextrose Agar (PDA) plates acidified with 10% tartaric acid. The minimum fungicidal concentration (MFC) of the lemon EO was determined via microdilution in 96-well plates using serial dilutions of the EO, Tween 80, and YPG broth, ranging from 8% to 0.125% [19].

Following dilution, 10 µL of the fungal inoculum was added to each well, and the plates were incubated at 30 °C for 48 h. Subsequently, aliquots from each well were subcultured onto acidified PDA plates and incubated at 30 °C for another 48 h. The MFC was defined as the lowest concentration that prevented visible fungal growth or resulted in fewer than 3 colony-forming units (CFU). Additionally, the disk diffusion method was applied for all EOs on acidified PDA plates following incubation at 30 °C for 48 h.

### 2.5. Preparation of Potato Starch and Citrus Essential Oil Bioactive Films

The experimental design comprised a total of eleven distinct film treatments, including a commercial cellophane film used as a reference control (CF) and a neat potato starch matrix without additives (PSF). The remaining nine active treatments were established by incorporating three types of citrus peel essential oils (lemon, orange, and tangerine) into the starch matrix, each evaluated at three concentration levels: 0.5%, 1.0%, and 2.0% (*w*/*w*). To standardize the nomenclature, active formulations were coded based on the starch matrix (PSF); the specific essential oil type, lemon (LEO), orange (OEO), or tangerine (TEO); and their respective mass fractions. Consequently, the treatments were designated as PSF-LEO (0.5%, 1%, 2%), PSF-OEO (0.5%, 1%, 2%), and PSF-TEO (0.5%, 1%, 2%). The incorporation levels of citrus essential oils (0.5, 1.0 and 2.0%, *w*/*w*) were selected based on the preliminary antifungal screening of the free oils and on concentration ranges previously reported for starch-based active films containing citrus essential oils [20,21].

The polymeric films were synthesized using the solvent casting method with adaptations [22]. The film-forming solution (FFS) was prepared using an aqueous mixture of potato starch (5% *w*/*v*), glycerol (1.5% *w*/*v*) as a plasticizer, and citric acid (0.7% *w*/*v*) as a crosslinking agent. Initially, the starch was dispersed in distilled water and homogenized under constant stirring at room temperature for 20 min. Subsequently, glycerol (Isofar, Duque de Caxias, RJ, Brazil) and citric acid (Arcolor, São Paulo, SP, Brazil) were added to the dispersion. The mixture was then heated to 80 °C under continuous stirring to induce starch gelatinization. The temperature during the FFS preparation was strictly monitored using a digital probe thermometer (Prolab, São Paulo, SP, Brazil). Once gelatinized, the FFS was allowed to cool down to 40 °C. At this stage, the different concentrations of lemon, orange, or tangerine essential oils (0.5%, 1.0%, and 2.0% *w*/*w*) were incorporated under vigorous stirring at 900 rpm for 15 min.

Prior to incorporation, the essential oils were pre-emulsified by adding Tween 80 (5% *w*/*w*) as a surfactant, followed by vortex homogenization for 5 min. The mixture was then subjected to ultrasonication in a sonicator bath (Prolab, São José dos Pinhais, PR, Brazil) operating at 1000 W and 30 °C for 40 min to promote droplet size reduction [23]. After this treatment, for each formulation, 98 g of the film-forming solution was uniformly cast into polypropylene trays (25 × 16 cm), corresponding to a casting density of approximately 0.245 g cm^−2^. The films were dried in a forced-air oven at 50 °C for 42 h and subsequently peeled from the casting trays and conditioned for further characterization.

### 2.6. Evaluation of the Antimicrobial Activity of the Bioactive Films

The antibacterial and antifungal activities of the developed starch films were evaluated using the agar disk diffusion method, adapted from the protocols described in Section 2.3 and Section 2.4. Instead of filter paper disks, the square film specimens were cut into dimensions of 1 × 1 cm and transferred aseptically using sterile stainless-steel tweezers onto the center of the Petri dishes. The plates had been previously inoculated with the respective target microorganisms: *Escherichia coli*, *Staphylococcus aureus*, and *Aspergillus niger*. Following the incubation periods specified for each microbial class, the plates were visually inspected for the presence of translucent inhibition zones around and beneath the film samples. The clear zones of growth inhibition indicated a positive antimicrobial effect, whereas the absence of such zones denoted a lack of bioactivity.

### 2.7. Characterization of the Starch-Based Bioactive Films

Based on the preliminary antimicrobial screening, only the films containing 1 and 2% (*w*/*w*) essential oils were selected for XRD, SEM, FTIR, TGA/DTG, transparency, biodegradation, and phytotoxicity analyses.

#### 2.7.1. Thickness

Film thickness was determined by measuring twelve random positions across each film sample using a digital micrometer caliper (MTX, Digital Caliper, Shanghai, China). The average values were used to normalize subsequent physical and mechanical characterizations.

#### 2.7.2. Color Analysis

The surface color of the films was evaluated using a colorimeter (Delta Color, São José dos Campos, SP, Brazil) under the CIELAB D_65_ illuminant system with a 10° observation angle [24]. The film specimens were placed over a standard white reference plate (L_0_*, a_0_*, b_0_*). The total color difference (ΔE*) was calculated using Equation (3).

ΔE* = [(ΔL*)^2^ + (Δa*)^2^ + (Δb*)^2^]^1/2^ (3)

where ΔL*, Δa*, and Δb* represent the variations between the color parameters of the active film formulations and the standard white background plate (L* ranges from 0 [black] to 100 [white]; a* ranges from green [−a*] to red [+a*]; and b* ranges from blue [−b*] to yellow [+b*]).

#### 2.7.3. Transparency

Light transmittance was evaluated at a wavelength of 600 nm using a UV–Vis spectrophotometer (KASVI, Pinhais, PR, Brazil) in accordance with ASTM D1746-15 [25]. Rectangular specimens (45 × 9 mm) of selected formulations (PSF, PSF-LEO 1%, PSF-LEO 2%, PSF-TEO 1%, and PSF-TEO 2%) were cut and positioned perpendicularly to the light beam path inside the cuvettes. Film transparency (T_600_) was calculated based on the relationship between light transmittance and film thickness, according to Equation (4).

T_600_ = [log_10_ (%*T*)]/b (4)

where T is the transmittance fraction and b is the average film thickness (mm).

The results were expressed as A_600_. Film thickness for this assay was measured at three distinct points using a digital micrometer, and all analyses were performed in triplicate.

#### 2.7.4. Scanning Electron Microscopy (SEM)

The surface and cross-sectional morphologies of the selected films (PSF, PSF-LEO 1%, PSF-LEO 2%, PSF-TEO 1%, and PSF-TEO 2%) were examined using a field-emission scanning electron microscope (FE-SEM; Tescan, model Mira 4, Brno, Czech Republic) equipped with a high-brightness Schottky field-emission gun (FEG) source. Dried film samples were cut, mounted onto the sample holder using conductive double-sided adhesive tape, and coated with a thin layer of gold to ensure adequate electrical conductivity. Micrographs of the surface and fractured cross-sections were recorded under high-vacuum conditions at an accelerating voltage of 5 kV.

#### 2.7.5. Water Content, Swelling Degree, and Water Solubility

The physical interactions between the polymeric matrices and water were assessed by measuring the water content (WC), degree of swelling (DS), and water solubility (WS) [26]. Square film specimens (2 × 2 cm) were cut and weighed to determine the initial mass (m_1_). The samples were then dried in an oven at 105 °C for 24 h to determine the dry mass (m_2_). Subsequently, the dried films were immersed in 30 mL of distilled water inside Falcon tubes and maintained at 25 °C for 24 h. The samples were removed, gently blotted with filter paper to eliminate surface water, and weighed immediately to obtain the swollen mass (m_3_). Finally, the films were dried again at 105 °C for 24 h to determine the final dry mass (m_4_). The WC (%), DS (%), and WS (%) values were calculated using Equations (5), (6), and (7), respectively.

WC (%) = [(m_1_ − m_2_)/m_1_] × 100 (5)

DS (%) = [(m_3_ − m_2_)/m_2_] × 100 (6)

WS (%) = [(m_2_ − m_4_)/m_2_] × 100 (7)

#### 2.7.6. X-Ray Diffraction (XRD)

The crystalline structure of potato starch (PS) and the films (PSF, PSF-LEO 1%, PSF-LEO 2%, PSF-TEO 1%, and PSF-TEO 2%) was investigated by X-ray diffraction (Shimadzu, model XRD-6100, Kyoto, Japan). The analysis was conducted using a copper X-ray (CuKα radiation, ƛ = 1.540 Å) operating at 40 kV and 30 mA. Scanning was performed at a continuous rate of 2°/min over a 2Ө angular range from 5° to 40°.

The crystallinity index (CrI) of the native potato starch and starch-based films was determined from the X-ray diffraction (XRD) patterns according to the method proposed by Nara et al. [27]. The crystalline and amorphous areas of each diffractogram were integrated using OriginPro 2024 software (OriginLab Corporation, Northampton, MA, USA), and the crystallinity index was calculated using Equation (8):

(8) $$\mathrm{CrI}\left(\%\right)=\frac{A_{c}}{A_{c}+A_{am}}\times 100$$

where CrI is the crystallinity index (%), $A_{c}$ is the integrated area corresponding to the crystalline regions, and $A_{am}$ is the integrated area of the amorphous region.

#### 2.7.7. Fourier Transform Infrared (FTIR) Spectroscopy

The chemical structures and functional groups of the developed films were analyzed using a Fourier Transform Infrared Spectrometer (Shimadzu, model IRAffinity-1S, Kyoto, Japan) equipped with an Attenuated Total Reflectance (ATR) accessory. Spectral data for each dehydrated sample were acquired in the wavenumber range of 4000 to 400 cm^−1^ with a resolution of 4 cm^−1^ and 32 scans. The resulting spectra were evaluated to investigate intermolecular interactions and potential structural changes within the starch matrix after the incorporation of the citrus essential oils.

#### 2.7.8. Thermogravimetric Analysis (TGA/DTG)

The thermal stability and degradation profiles of the formulations were determined via thermogravimetric analysis (TGA) and its derivative (DTG) using a thermal analyzer (Shimadzu, model TGA-51, Kyoto, Japan). Film samples (10 mg) were heated from 30 °C to 600 °C at a constant heating rate of 10 °C/min. The analysis was conducted in an inert nitrogen atmosphere with a constant flow rate of 50 mL/min to prevent oxidative degradation. The onset temperature (T*_onset_*), the temperature of maximum degradation rate (*T_max_*), and the final residual mass were monitored to characterize the thermal behavior of the matrices.

#### 2.7.9. Mechanical Properties

The mechanical performance of the films, specifically tensile strength (TS) and elongation at break (EB), was measured using a Universal Testing Machine (Model 3365, Instron Corporation, Norwood, MA, USA) in strict accordance with the ASTM D882-18 standard [28]. Film samples were cut into rectangular strips with dimensions of 25 × 100 mm. The crosshead speed was set to 2 mm/s, utilizing a 50 kgf load cell and an initial grip separation distance of 50 mm. All mechanical tests were performed in quintuplicate (n = 5) at room temperature [24].

#### 2.7.10. Soil Biodegradability Analysis

The film biodegradability was evaluated according to Reichert et al. [29] and Costa [24], following the ASTM G160-03 standard [30]. Triplicates of rectangular film specimens (2 × 3 cm; formulations PSF, PSF-LEO 1%, PSF-LEO 2%, PSF-TEO 1%, and PSF-TEO 2%) were dried at 60 °C to constant mass (W_0_). CF was used as a reference control. The samples were placed inside double-layered nylon mesh bags and buried at a depth of 3.5 cm in 5 L plastic containers filled with natural organic soil at pH = 6.0.

The assay was conducted under ambient conditions (December 2024 to January 2025), with an average temperature of 27.3 °C and 80% relative air humidity (according to meteorological data provided by the National Institute of Meteorology (INMET), Brazil). Soil moisture was maintained at approximately 40% by adding distilled water every 48 h, and soil parameters were monitored using a multi-parameter meter (G.C Max, China). Samples were recovered at 0, 5, 10, 15, 30, and 45 days. At each interval, the remaining film fragments were removed, washed with distilled water, blotted with absorbent paper, and dried at 60 °C until constant dry weight was achieved (W_f_). The weight loss percentage (WL) was determined using Equation (9).

%WL = [(W_0_ − W_f_)/W_0_] × 100 (9)

where WL is the percentage of weight loss, W_0_ is the initial dry weight of the film before soil burial (g), and W_f_ is the dry weight of the recovered film after soil burial and drying to constant weight at each sampling interval (g).

#### 2.7.11. Phytotoxicity Assay

The phytotoxicity of the degraded film residues was evaluated to investigate the potential release of toxic compounds that could hinder plant development [29]. Coriander seeds (*Coriandrum sativum*) were used as the biological model. The assay was conducted in plastic germination trays, where 1.0 g of each film formulation (PSF, PSF-LEO 1%, PSF -LEO 2%, PSF-TEO 1%, and PSF-TEO 2%) and the CF was crushed and thoroughly mixed with a commercial soil substrate (Carolina Soil do Brasil Ltda, Santa Cruz do Sul, RS, Brazil). For each treatment, three replicates containing three seeds each were planted. Plant growth was monitored for 20 days. Subsequently, the two largest seedlings from each cell were harvested to determine the average fresh and dry biomass, assessing the influence of the polymeric film residues on seed germination and early plant development.

### 2.8. Statistical Analysis

Experimental data were expressed as means and standard deviation. The data compliance with parametric assumptions was verified; subsequently, one-way Analysis of Variance (ANOVA) followed by Tukey’s post hoc test was applied for parametric datasets. For non-parametric datasets, the Mann–Whitney and Dunn’s post hoc tests were utilized. All statistical comparisons were performed at the 5% significance level (α = 0.05) using PAST software (version 4.07b) [31]. Additionally, Principal Component Analysis (PCA) was executed to multivariate-classify and select the optimized bioactive film formulations.

## 3. Results

### 3.1. Extraction Yield and Physical Properties of Citrus Essential Oils

The extraction parameters, yield, and density of the essential oils (EOs) obtained from the peels of lemon (*Citrus latifolia* Tanaka), orange (*Citrus sinensis*), and tangerine (*Citrus reticulata*) via hydrodistillation are summarized in Table 1.

Significant variations in extraction yields were observed among the citrus species. Under a standardized fresh biomass of 300 g, *C. sinensis* (orange) peel produced the highest essential oil volume (5.40 mL), resulting in an extraction yield of 1.80% (*v*/*w*). This yield was more than double those obtained for *C. latifolia* (lemon; 0.84% *v*/*w*) and *C. reticulata* (tangerine; 0.70% *v*/*w*). These discrepancies are primarily attributed to the anatomical distribution and density of oil glands (secretory cavities) in the flavedo of each citrus species, as well as seasonal and physiological factors of the fruits.

Regarding physical properties, the density of the extracted oils varied within a narrow range (0.839 to 0.847 g/cm^3^), which is consistent with light, volatile terpene-rich fractions. *C. reticulata* (tangerine) essential oil exhibited the highest density (0.847 g/cm^3^), followed by *C. latifolia* (0.842 g/cm^3^) and *C. sinensis* (0.839 g/cm^3^). The lower density of orange essential oil is typically correlated with a higher concentration of monoterpene hydrocarbons, particularly d-limonene, which possesses a lower specific gravity compared with oxygenated sesquiterpenes and coumarins that may be more abundant in tangerine peel oil. These physical characteristics, particularly density and hydrophobic character, play a crucial role in the subsequent interfacial dispersion of the oils within the hydrophilic starch-based polymeric matrix.

### 3.2. Chemical Composition of Citrus Essential Oils via GC-MS

The volatile chemical profiles of the essential oils (EOs) extracted from lemon (*C. latifolia*), orange (*C. sinensis*), and tangerine (*C. reticulata*) peels were determined via GC-MS. The comparative volatile chemical profiles and relative abundance of the phytocompounds identified in the extracted essential oils are summarized in Table 2.

The volatile profiles of all three citrus EOs were dominated by monoterpene hydrocarbons, with limonene being identified as the primary active constituent. However, the concentration of this major terpene varied substantially among the species. *C. sinensis* (orange) EO exhibited the highest limonene purity (95.78%), followed by *C. reticulata* (tangerine; 76.22%) and *C. latifolia* (lemon; 57.79%).

For lemon EO, the lower relative abundance of limonene was compensated for by significant amounts of other valuable monoterpenes, such as β-pinene (15.96%) and γ-terpinene (16.00%). In tangerine EO, the second most abundant component was γ-terpinene (9.85%), accompanied by o-cymene (4.14%) and α-pinene (2.84%).

The presence of these distinct volatile compounds, which differ in molecular size, steric hindrance, and volatility, directly influences their physical behavior inside the carbohydrate polymer. While a highly homogeneous monoterpene phase, such as orange oil, tends to form distinct hydrophobic clusters, a more complex blend of terpenes, as seen in lemon and tangerine oils, can lead to different interfacial packing and molecular mobility when dispersed within the gelatinized starch matrix.

### 3.3. Antibacterial Efficacy and Minimum Inhibitory Concentration (MIC) of Citrus Essential Oils

**Figure 1:**
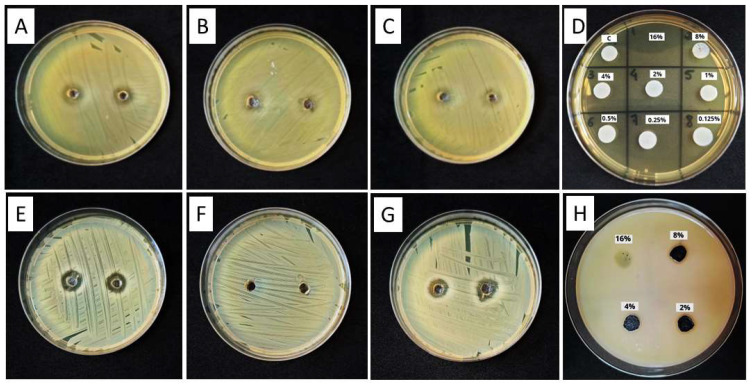
Antibacterial profiles of citrus essential oils. Agar disk diffusion zones (**A**,**C**,**E**,**G**) and min-imum inhibitory concentration (MIC) of lemon (*Citrus latifolia*) essential oil (**D**,**H**) against *Escherichia coli* (**A**–**D**) and *Staphylococcus aureus* (**E**–**H**). Legend: (**A**,**D**,**E**,**H**) Lemon (*Citrus latifolia*) essential oil; (**B**,**F**) Pera orange (*Citrus sinensis*) essential oil; (**C**,**G**) Ponkan tangerine (*Citrus reticulata*) essential oil.

The in vitro antibacterial activities of the lemon (*C. latifolia*), orange (*C. sinensis*), and tangerine (*C. reticulata*) essential oils (EOs) against Gram-negative *Escherichia coli* and Gram-positive *Staphylococcus aureus* were quantitatively evaluated using the agar disk diffusion and minimum inhibitory concentration (MIC) assays. The experimental results are summarized in Table 3 and Figure 1.

In the agar disk diffusion assay, significant differences in the inhibition zones were observed among all evaluated groups (*p* < 0.05). Against Gram-negative *E. coli*, lemon EO exhibited the largest zone of inhibition (13.23 ± 0.15 mm), followed by tangerine EO (12.18 ± 0.10 mm) and orange EO (11.12 ± 0.08 mm).

For Gram-positive *S. aureus*, lemon and tangerine EOs showed substantial inhibitory effects, producing distinct inhibition zones of 13.25 ± 0.13 mm and 12.31 ± 0.10 mm, respectively, which were statistically different from each other (*p* < 0.05). Conversely, no visible zone of inhibition (ND) was recorded for orange EO against *S. aureus*.

In the MIC assay, only lemon EO presented a detectable minimum inhibitory concentration within the tested concentration range. Specifically, lemon EO achieved an MIC of 16.0% (*v*/*v*) against both *E. coli* and *S. aureus*. Under the same test conditions, both orange and tangerine EOs failed to completely inhibit bacterial growth at concentrations up to 16.0% (*v*/*v*), indicating that higher free-oil concentrations are required.

### 3.4. Antifungal Activity of Citrus Essential Oils

The antifungal profiles of the citrus peel essential oils and the ketoconazole control against *Aspergillus niger* and bread mold fungi are summarized in Table 4 (Supplementary Tables S1–S3). In the agar diffusion assay, statistically significant variations in the zones of inhibition against *Aspergillus niger* were observed among all tested groups (*p* < 0.05). The positive control, ketoconazole, yielded the largest inhibition halo (45.35 ± 4.91 mm), followed by tangerine EO (27.32 ± 3.01 mm) and orange EO (22.44 ± 2.42 mm). Conversely, lemon EO did not produce a measurable inhibition zone (0.00 ± 0.00 mm) against this specific fungal strain.

For the bread mold fungal screening, orange EO promoted a distinct radial inhibition zone of 23.41 ± 0.00 mm, while lemon EO resulted in a zone of 16.59 ± 0.00 mm. Tangerine EO did not induce a clear, measurable zone of radial clearing, displaying instead a prominent fungistatic activity across the application matrix area.

Regarding the quantitative MIC evaluation, both lemon and orange EOs exhibited minimum inhibitory concentrations at a baseline of 2.0% (*v*/*v*), which corresponds to mass concentrations of 20.2 and 20.0 mg·mL^−1^, respectively. For tangerine EO, the minimum inhibitory concentration required to suppress active fungal proliferation was established at 4.0% (*v*/*v*), corresponding to a mass concentration of 33.8 mg/mL.

### 3.5. Antimicrobial Activity of Developed Biofilms

The antimicrobial screening of the potato starch films incorporated with different concentrations (0.5, 1 and 2% *v*/*v*) of citrus peel essential oils against *Staphylococcus aureus*, *Escherichia coli*, and *Aspergillus niger* is summarized in Table 5 (Supplementary Tables S4–S6).

**Figure 2:**
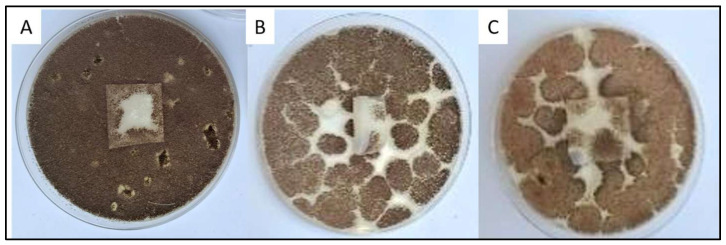
Fungistatic activity of potato starch-based films: PSF (**A**) and with the addition of 2% LEO (**B**) and 2% TEO (**C**).

Regarding the antibacterial trials, the control (PSF) displayed no inhibitory activity against any tested microorganisms. For the Gram-negative bacteria *E. coli*, all incorporated active films remained completely inactive across the entire concentration range. Conversely, a selective bacteriostatic response against Gram-positive *S. aureus* was observed exclusively for the films blended with lower concentrations of lemon essential oil, specifically PSF–LEO 0.5% and PSF–LEO 1.0%. Higher concentrations of LEO (2.0%), as well as all formulations containing OEO and TEO, failed to inhibit *S. aureus* proliferation within the film contact area (Figure 2).

In the antifungal screening against *A. niger*, distinct fungistatic effects were recorded depending on the oil source and loading concentration. The active film loaded with 2.0% lemon essential oil (PSF–LEO 2.0%) successfully inhibited fungal growth. Furthermore, the tangerine essential oil-loaded films demonstrated active fungistatic protection at both intermediate and higher replacement levels, specifically at PSF–TEO 1.0% and PSF–TEO 2.0%, visibly restricting target fungal development. No antifungal response was observed for any of the orange essential oil (OEO) film formulations.

### 3.6. Physical and Optical Characterization of the Active Biofilms

#### 3.6.1. Thickness, Color Parameters, and Opacity/Transparency

The physical dimensions (thickness) and optical properties (CIELab color coordinates L*, a*, and b*; total color difference ΔE; and transparency) of the neat potato starch film (PSF), the active formulations, and the CF are summarized in Table 6.

The incorporation of different citrus essential oils (EOs) induced significant modifications in film thickness (*p* < 0.05). The highest thickness values were recorded for formulations containing tangerine essential oil at higher loading levels, specifically PSF–TEO (0.21 mm) and PSF–TEO (0.17 mm). Conversely, the addition of orange essential oil (OEO) at all tested concentrations resulted in significantly lower thickness parameters compared with the other active matrices and the neat PSF matrix (*p* < 0.05). No statistically significant differences in thickness were detected between the neat PSF control and the formulations loaded with lemon essential oil at higher levels (PSF–LEO 1.0% and PSF–LEO 2.0%). However, lower oil loadings for specific groups (PSF–LEO 0.5%, PSF–TEO 0.5%, PSF–OEO 0.5%, PSF–OEO 1.0%, and PSF–OEO 2.0%) promoted a significant reduction in overall film thickness when contrasted with the pure starch matrix (*p* < 0.05). The CF control presented the lowest absolute thickness values among all evaluated materials.

Regarding the colorimetry, the neat polysaccharide matrix exhibited significantly higher lightness values (L* = 61.24) than the CF matrix (L* = 31.23, *p* < 0.05). The immobilization of all volatile oil fractions reduced the L* parameter of the films relative to the pure PSF matrix. For the chromatic coordinate a*, no statistically significant differences were observed among CF, neat PSF, PSF–LEO, and PSF–TEO. Conversely, the remaining active formulations displayed significantly more negative values compared with the pure starch control, indicating a displacement toward the green spectrum.

For the yellowish/blueish coordinate b*, pure starch control displayed positive values (0.54), whereas the CF matrix exhibited negative values (−0.754). Among the active matrices, only the PSF–TEO formulation maintained a value statistically similar to the pure PSF control (*p* > 0.05). For the remaining formulations, essential oil incorporation did not cause significant variations in the parameter relative to the CF baseline. The total color difference (∆E) significantly decreased in all oil-loaded biofilms when compared with both PSF and CF controls (*p* < 0.05), though no significant differences were observed among the different citrus oil sources and concentrations.

**Figure 3:**
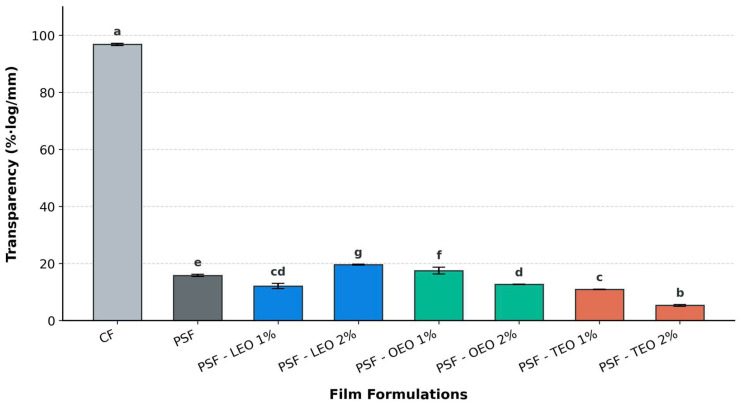
Transparency of potato starch-based films incorporated with citrus peel essential oils. Legend: Different superscript letters on the bars indicate statistically significant differences (*p* < 0.05) determined by Tukey’s post-hoc test.

**Figure 4:**
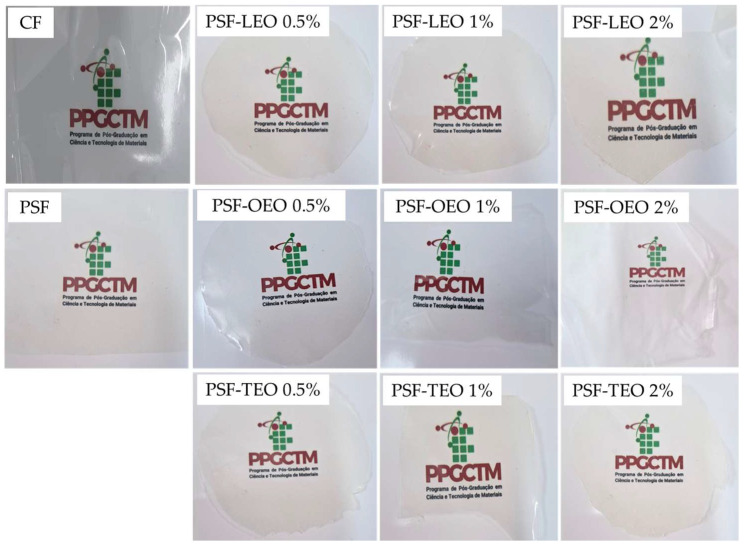
Visual appearance of potato starch-based films with and without the addition of citrus essential oils.

In terms of optical barriers. the formulations PSF–OEO 1.0% and PSF–LEO 2.0% demonstrated significantly higher transparency values than the neat PSF control (*p* < 0.05). In contrast, the addition of 2.0% tangerine essential oil (PSF–TEO 2.0%) reduced light transmission, yielding the lowest transparency value among all samples. In general, the starch-based films displayed lower overall transparency parameters than the reference commercial control (CF), as visually represented in Figure 3 and Figure 4.

#### 3.6.2. Water Content, Water Solubility, and Degree of Swelling

The water affinity parameters of the developed matrices, encompassing water content (WC), water solubility (WS), and degree of swelling (DS), are summarized in Table 7.

The incorporation of citrus essential oils (EOs) did not significantly affect the water content (WC) of the starch-based films (*p* > 0.05). Although slight variations in the mean values were observed among the formulations, no statistically significant differences were detected, indicating that neither the type nor the concentration of the essential oils altered the moisture retention capacity of the polymeric matrix.

Similarly, the water solubility (WS) of the films was not significantly influenced by the incorporation of citrus essential oils (*p* > 0.05). The WS values ranged from 23.33 to 37.78%, demonstrating that the incorporation of the different essential oils did not compromise the integrity of the starch matrix under the immersion conditions evaluated.

In contrast, the degree of swelling (DS) was significantly affected by the incorporation of citrus essential oils (*p* < 0.05). Films containing higher concentrations of essential oils, particularly PSF–LEO 2% (111.11 ± 19.25%) and PSF–TEO 1% (112.22 ± 10.72%), exhibited significantly greater swelling than the neat starch film (PSF, 55.19 ± 5.01%), whereas the remaining formulations showed intermediate behavior and did not differ statistically from either the control or the formulations with the highest swelling values. These results suggest that higher essential oil loadings increased the water absorption capacity of the starch matrix, probably by promoting a less compact polymeric network and facilitating water diffusion into the films.

#### 3.6.3. Scanning Electron Microscopy (SEM)

**Figure 5:**
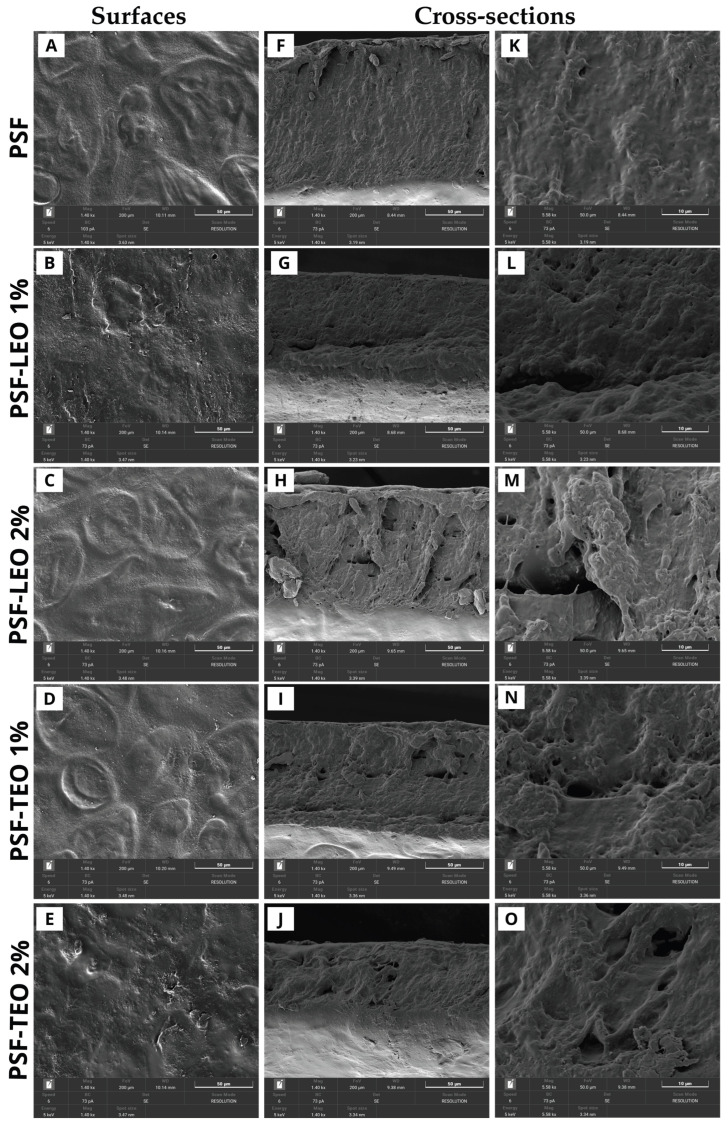
SEM micrographs showing the surface topography and cross-sectional morphology of neat potato starch films (PSF), and active formulations incorporated with citrus peel essential oils (LEO, and TEO). Legend: (**A**–**E**) surface micrographs with magnification of 5.58 kx (10 μm); (**F**–**J**) cross-sectional micrographs with magnification of 1.4 kx (50 μm); (**K**–**O**) cross-sectional micrographs with magnification of 5.58 kx (10 μm), using an accelerating voltage of 5 kV.

The surface and cross-sectional microstructures of the developed starch-based matrices and control groups are displayed in Figure 5. The neat potato starch film (PSF) and the reference presented a smooth, homogeneous, and continuous matrix baseline, characterized by a cohesive polymeric network free of fractures or ungelatinized starch granules.

Conversely, the immobilization of citrus peel essential oils (EOs) induced clear structural modifications. The active formulations exhibited an increase in surface roughness and a distinctly discontinuous cross-sectional morphology. Micrographs of the oil-loaded matrices revealed a widespread distribution of micro-voids, pores, and spherical cavities trapped within the hydrophilic carbohydrate backbone. These microstructural gaps became progressively more pronounced at higher active agent loadings (1.0% and 2.0% *v*/*v*), corresponding to the micro-droplet locus sites of the lipid-phase separation fixed during film drying. No structural failure planes or deep cracks were observed across any of the active formulations. 

#### 3.6.4. X-Ray Diffraction (XRD)

**Figure 6:**
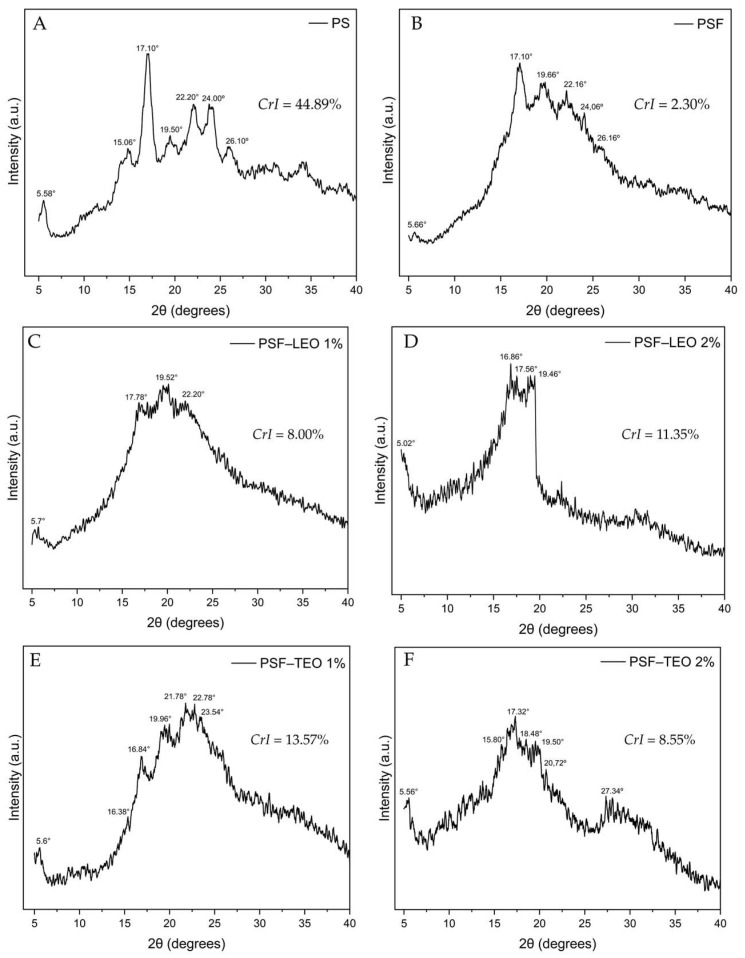
X-ray diffraction patterns and crystallinity index (CrI) of native potato starch and starch-based bioactive films incorporated with citrus essential oils. Legend: (**A**) Native potato starch (PS); (**B**) potato starch film (PSF); (**C**) PSF incorporated with 1% lemon essential oil (PSF-LEO 1%); (**D**) PSF incorporated with 2% lemon essential oil (PSF-LEO 2%); (**E**) PSF incorporated with 1% tangerine essential oil (PSF-TEO 1%); and (**F**) PSF incorporated with 2% tangerine essential oil (PSF-TEO 2%).

The XRD diffractograms of the native potato starch and the developed starch-based films are displayed in Figure 6. The native potato starch (PS) (Figure 6A) exhibited the characteristic B-type crystalline pattern, with well-defined diffraction peaks centered at 2θ = 5.58°, 15.06°, 17.10°, 19.50°, 22.20°, 24.00°, and 26.10°, in agreement with the diffraction profile commonly reported for potato starch [27,32]. After film formation, the neat potato starch film (PSF) (Figure 6B) presented a marked reduction in the intensity of the crystalline reflections due to the thermal gelatinization process conducted at 80 °C in the presence of glycerol and 0.7% citric acid. The diffraction profile was characterized by broad reflections centered at approximately 2θ = 17.10°, 19.66°, 22.16°, 24.06°, and 26.16°, indicating the extensive disruption of the native B-type crystalline structure and the predominance of an amorphous starch matrix.

The incorporation of citrus peel essential oils modulated the intensity and distribution of the diffraction planes depending on the volatile source and concentration. For the lemon essential oil formulations, the film loaded with 1.0% LEO (Figure 6C) exhibited broad diffraction peaks centered at approximately 2θ = 17.78°, 19.52°, and 22.20°, indicating partial molecular rearrangement after gelatinization. Increasing the essential oil concentration to 2.0% LEO (Figure 6D) resulted in more defined crystalline reflections at 2θ = 16.86°, 17.56°, and 19.46°, together with the reappearance of a low-angle reflection at approximately 5.02°, suggesting partial recovery of the B-type crystalline organization.

Regarding the tangerine essential oil group, the film incorporated with 1.0% TEO (Figure 6E) exhibited diffraction peaks centered at approximately 2θ = 16.38°, 16.84°, 19.96°, 21.78°, 22.78°, and 23.54°, indicating a greater degree of structural organization than the control film. Conversely, the formulation containing 2.0% TEO (Figure 6F) presented reflections at approximately 2θ = 5.56°, 15.80°, 17.32°, 18.48°, 19.50°, 20.72°, and 27.34°, demonstrating that the higher concentration of essential oil modified the crystalline arrangement of the starch matrix while preserving reflections associated with the native B-type polymorphism. The reappearance of the low-angle reflections around 5–6° (2θ) in the PSF–LEO 2% and PSF–TEO 2% films further support the partial recovery of B-type crystalline domains after essential oil incorporation, although the overall diffraction profiles remained predominantly amorphous.

The crystallinity index (CrI) was estimated from the XRD diffractograms to quantify the structural changes induced by gelatinization and essential oil incorporation. Native potato starch exhibited a CrI of 44.89%, confirming its well-defined B-type crystalline organization. After gelatinization and film formation, the crystallinity index decreased sharply to 2.30% for the control starch film (PSF), demonstrating the extensive disruption of the native crystalline structure. The incorporation of citrus essential oils promoted a partial recovery of structural organization, increasing the CrI to 8.00% and 11.35% for PSF–LEO 1% and PSF–LEO 2%, respectively, and to 13.57% and 8.55% for PSF–TEO 1% and PSF–TEO 2%, respectively.

These results suggest that the essential oils influenced the molecular rearrangement of starch chains during film formation, promoting localized crystalline organization while maintaining the predominantly amorphous character of the polymeric matrix. The highest crystallinity observed for PSF–TEO 1% indicates that moderate concentrations of tangerine essential oil favored chain rearrangement, whereas increasing the concentration to 2% reduced the crystallinity, suggesting that higher oil contents may have partially hindered the regular packing of starch chains within the polymeric matrix. Despite this partial recrystallization, the crystallinity indices of all bioactive films remained substantially lower than that of the native potato starch, confirming that thermal gelatinization was the predominant factor governing the structural organization of the developed films.

#### 3.6.5. Fourier Transform Infrared (FTIR) Spectroscopy

**Figure 7:**
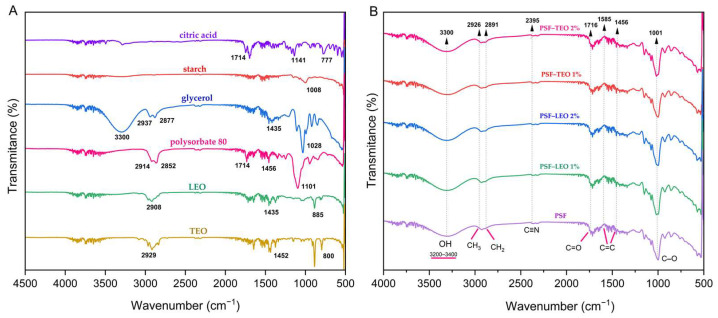
Fourier Transform Infrared (FTIR) spectra of: (**A**) the pure, raw components utilized in the development of the active polymeric films and (**B**) the formulated potato starch-based films with and without the incorporation of citrus peel essential oils.

The FTIR spectra of the pure, raw materials used in the film formulations are shown in Figure 7, along with the infrared spectra of the developed potato starch-based films, with and without the incorporation of citrus peel essential oils.

The analysis of the active and control matrices revealed the presence of characteristic absorption bands associated with their major constituent components. A broad and intense absorption band observed in the 3500–3200 cm^−1^ region was attributed to the stretching vibrations of hydroxyl groups (–OH), which are correlated with the polyol structures of starch and glycerol, as well as the carboxylic groups of citric acid. The asymmetric stretching vibrations of aliphatic C–H bonds were recorded at 2926 cm^−1^, representing the alkane structures present in the polysaccharide backbone and the terpene fractions of the essential oils.

The carbonyl stretching vibration (C=O) was identified at 1716 cm^−1^, confirming the presence of ester and carboxylic acid functionalities from the crosslinker and surfactant agents. Additionally, carbon–carbon double bond (C=C) aromatic/alkene stretching was recorded at 1585 cm^−1^ and 1456 cm^−1^. Lastly, the characteristic carbohydrate fingerprints, associated with the stretching of C–O–C ether groups, were observed at 1001 cm^−1^. The infrared profiles of the active films loaded with different concentrations of lemon essential oil (PSF–LEO) and tangerine essential oil (PSF–TEO) demonstrated overlapping spectra with no shifts in the baseline absorption bands or the emergence of new chemical peaks compared with the neat PSF matrix.

#### 3.6.6. Thermogravimetric Analysis (TGA/DTG)

**Figure 8:**
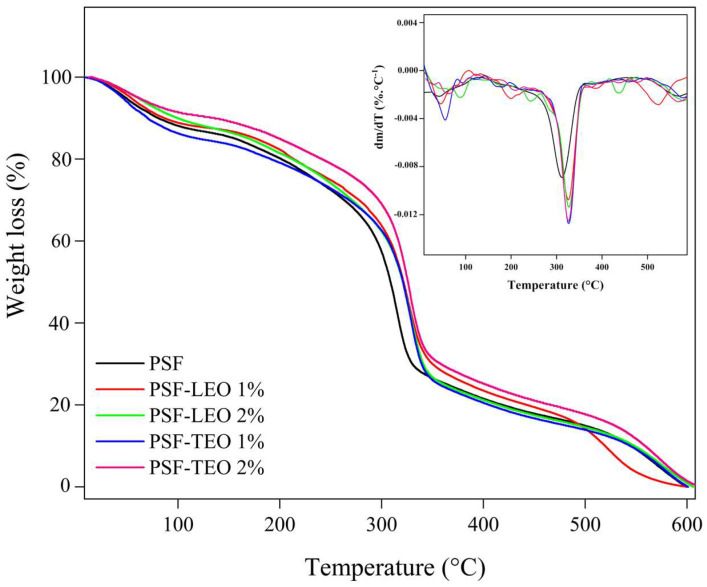
Thermal stability profiles of neat potato starch films (PSF) and active formulations incorporated with citrus peel essential oils.

The thermogravimetric (TGA) profiles and their respective derivative (DTG) curves detailing the thermal stability and degradation behavior of the developed starch-based active films are displayed in Figure 8. All evaluated formulations exhibited a multi-stage weight loss profile spanning across distinct thermal decomposition windows.

The initial weight loss stage, occurring from baseline room temperature up to approximately 120 °C, corresponded to a mass reduction of roughly 10–15%, driven by the evaporation of free and weakly bound water molecules (moisture content) trapped within the hydrophilic polysaccharide networks. A second, minor decomposition step was recorded between 150 °C and 260 °C, attributed to the volatilization of the plasticizer agent (glycerol) alongside the thermal stripping of the volatile terpenoid fractions belonging to the immobilized lemon (LEO) and tangerine (TEO) essential oils.

The primary and most abrupt thermal degradation event took place within the 280 °C to 360 °C temperature range, centered around a sharp peak in the DTG curves (Figure 8) at approximately 325 °C. This major step accounted for the thermal depolymerization and breakdown of the structural carbohydrate backbones (amylose and amylopectin segments) of potato starch.

The incorporation of the active citrus essential oils shifted the degradation baseline profiles without modifying the core decomposition pathways. Specifically, higher active agent loading levels—such as the formulations incorporated with 2.0% essential oils (PSF–LEO 2% and PSF–TEO 2%)—exhibited a slight thermal stabilization effect, maintaining higher residual mass percentages across the main degradation window compared with the neat PSF matrix and the 1.0% oil-loaded formulations. Above 400 °C, a final, gradual weight loss stage was observed, corresponding to the slow carbonization and decomposition of organic residual char materials.

#### 3.6.7. Mechanical Properties

**Figure 9:**
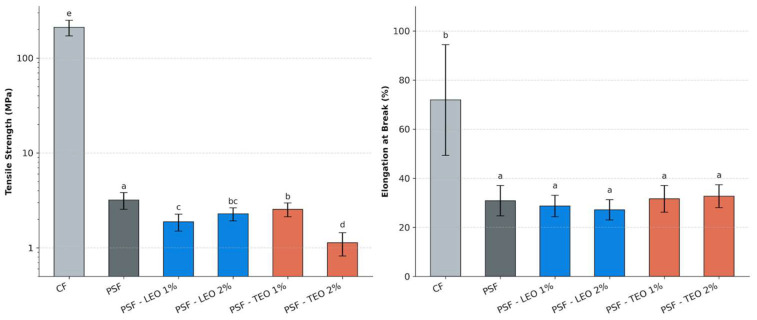
Mechanical performance parameters of commercial cellophane film (CF), neat potato starch films (PSF), and active films incorporated with lemon (LEO) and tangerine (TEO) essential oils. Legend: Different superscript letters on the bars indicate statistically significant differences (*p* < 0.05) determined by Tukey’s post-hoc test.

The mechanical performance of the developed starch-based matrices, indicating the impact of citrus peel essential oil incorporation on film integrity, is illustrated in Figure 9. The CF exhibited the highest mechanical resistance, reaching a TS value of 210.50 ± 39.61 MPa, which was statistically superior (*p* < 0.05) to all starch-based formulations. Among the biopolymer matrices, the neat potato starch film (PSF) displayed a maximum TS of 3.19 ± 0.64 MPa.

The incorporation of citrus peel essential oils significantly altered the tensile resistance depending on the active agent source and concentration. The addition of 1.0% lemon essential oil (PSF–LEO 1%) reduced the tensile profile to 1.88 ± 0.38 MPa, while an increase to 2.0% LEO (PSF–LEO 2%) resulted in a TS of 2.28 ± 0.36 MPa. Regarding the tangerine essential oil groups, the film loaded with 1.0% TEO (PSF–TEO 1%) maintained an intermediate tensile value of 2.55 ± 0.42 MPa, whereas scaling the oil loading up to 2.0% TEO (PSF–TEO 2%) induced the lowest absolute mechanical resistance among all evaluated groups, falling to 1.13 ± 0.31 MPa (*p* < 0.05).

In terms of flexibility and elongation at break (EAB), the CF reference displayed a high extensibility of 71.88 ± 22.57%. In contrast, the baseline polysaccharide matrix and the active essential oil-loaded films showed no statistically significant variations among themselves (*p* > 0.05). The EAB values remained statistically uniform across the neat PSF control (30.86 ± 6.16%) and the oil-incorporated groups, specifically PSF–LEO 1% (28.69 ± 4.37%), PSF–LEO 2% (27.14 ± 4.16%), PSF–TEO 1% (31.61 ± 5.47%), and PSF–TEO 2% (32.68 ± 4.67%).

#### 3.6.8. Biodegradability

**Figure 10:**
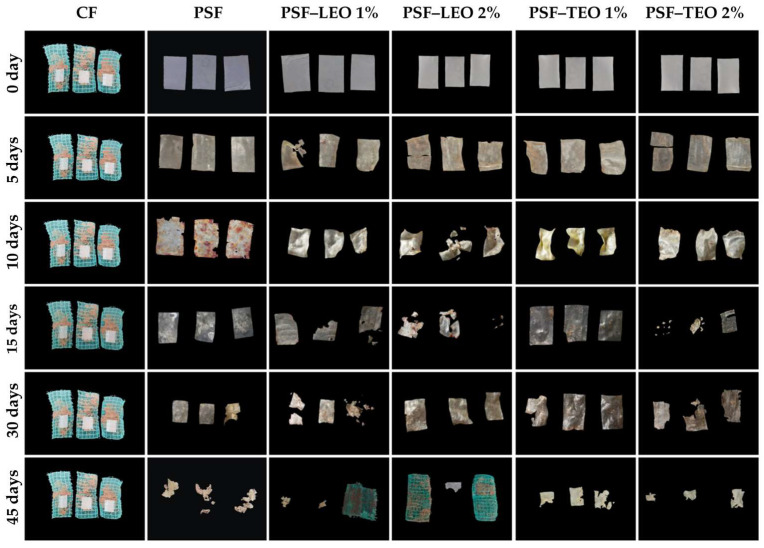
Biodegradability of commercial control and active potato starch-based films during the 45-day soil burial experiment.

The macrostructural degradation kinetics and physical appearance of the developed starch-based active matrices and the commercial reference group over a 45-day soil burial assay are illustrated in Figure 10. The corresponding quantitative mass-loss values recorded at intervals of 0, 5, 10, 15, 30, and 45 days are compiled in Table 8.

The statistical analysis revealed significant variations (*p* ≤ 0.05) in mass-loss percentages both as a function of exposure time and among the different formulations, as confirmed by Tukey’s post hoc test. The CF maintained structural integrity and exhibited negligible degradation or visual changes throughout the entire 45-day experimental timeframe.

Conversely, all starch-based films exhibited progressive, time-dependent degradation behavior. The mass loss-kinetics accelerated as a function of burial duration and was modulated by the incorporation and concentration of the citrus peel essential oils. Structural breakdown and fragmentation became visually apparent by day 5 for all biopolymer samples.

The most intensive degradation phase occurred between days 5 and 15. During this period, the incorporation of citrus essential oils influenced the degradation rate depending on both the type and concentration of the oil. Films containing 2% essential oil exhibited the highest mass losses after 15 days, reaching 79.2% for PSF–LEO 2% and 74.7% for PSF–TEO 2%, whereas the neat starch film (PSF) and PSF–TEO 1% showed lower mass losses of 39.1% and 48.7%, respectively. These results suggest that higher essential oil loadings may have increased the susceptibility of the starch matrix to water penetration and microbial attack, thereby accelerating the biodegradation process. This accelerated mass loss was consistent with the advanced fragmentation and severe structural erosion observed in the macrostructural images (Figure 10). By day 45, all starch-based films exhibited evident macroscopic deterioration. However, the extent of degradation differed among the formulations. The PSF–LEO films showed the highest mass losses (>90%), consistent with their extensive macroscopic disintegration, whereas the neat PSF and PSF–TEO films retained a greater proportion of their original mass despite exhibiting visible structural deterioration.

#### 3.6.9. Phytotoxicity Analysis

**Figure 11:**
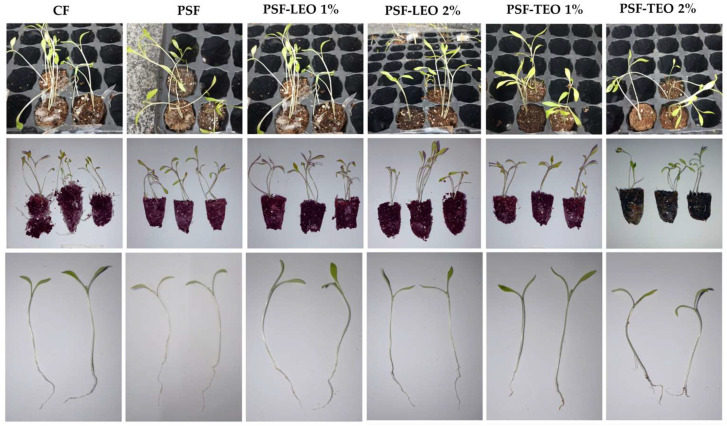
Phytotoxicity evaluation mapping of commercial cellophane film (CF), neat potato starch films (PSF), and active films incorporated with lemon (LEO) and tangerine (TEO) essential oils on coriander (*Coriandrum sativum*) seedlings after 20 days of growth.

The ecotoxicological impact of the developed starch-based active films and their degradation by-products on the environment was evaluated through a seedling development assay using coriander seeds (*Coriandrum sativum*). The biometric indicators, comprising macroscopic appearance, shoot/root growth uniformity, and biomass allocation after 20 days of planting, are presented in Figure 11. The corresponding quantitative fresh and dry mass yields are compiled in Table 9.

The statistical analysis performed via ANOVA confirmed that the presence of the polymeric matrices, including those formulated with high volumetric loadings of citrus peel essential oils, did not exert any inhibitory or statistically significant negative effects (*p* > 0.05) on the germination and physiological development of the plants. The fresh mass of the coriander seedlings varied within a narrow, statistically uniform baseline ranging from 0.07 ± 0.01 g (CF group) down to 0.04 ± 0.00 g (PSF–TEO 2% active group).

Furthermore, the dry matter accumulation remained perfectly constant at 0.01 ± 0.00 g across all evaluated experimental groups, confirming equal structural development. Visually, the seedlings grown in soil exposed to the neat PSF matrix and the active essential oil-loaded films (PSF–LEO and PSF–TEO fractions) exhibited healthy green shoots and elongated, well-branched root systems, matching the morphology observed in the CF reference group.

## 4. Discussion

The development of active and sustainable packaging has focused on incorporating bioactive compounds into hydrophilic polymeric matrices to confer functional properties, such as antimicrobial activity, and to improve the physical stability of the material. In this context, citrus fruit peels emerge as promising sources of essential oils rich in industrially relevant terpenes. The yields and densities obtained fell within the general variation range of 0.3% to 1.5% by mass stipulated by Silvestre and Pauletti [33] for citrus by-products. The extraction yield exceeded the averages reported by Simas et al. [34] and Ambriz-Pérez [35]. The density (0.881 g/mL) was close to the values found by Ambriz-Pérez [35] and Castro and Lima [36]. These fluctuations are governed by agroclimatic [37] and seasonal variations [38], and the fractions preserved their biological safety classification as GRAS substances [39].

GC-MS characterization confirmed the predominance of hydrocarbon monoterpenes, with emphasis on D-limonene, γ-terpineno, and β-pinene in Tahiti lemon peel (*Citrus latifolia* Tanaka), corroborating literature data [40,41]. The abundance of these molecules, also mapped by Teixeira et al. [42], consolidates such compounds as markers for the genus. Limonene acts as a safe aromatic additive [34,39], while γ-terpineno stands out for its active antimicrobial action.

The antimicrobial assays performed with the free essential oils provided a preliminary assessment of their intrinsic antimicrobial potential and served as a basis for selecting the incorporation levels used in the starch films. In biological susceptibility assays, the oils manifested action against the Gram-positive strain *Staphylococcus aureus*, meeting the positive criterion of Prabuseenivasan et al. [43]. However, under the classification scales of Semeniuc et al. [44] and Xand Neagu et al. [45], the obtained diameter is classified as very low inhibitory effect (<10 mm). This discrete performance aligns with the profile mapped by Altun and Yapici [46], inferring lower efficacy of hydrocarbon terpenes compared with oils rich in aldehydes and cinnamic phenols. Superior results were reported by Tran et al. [47], reinforcing the selective sensitivity of Gram-positive bacteria over Gram-negative ones and the influence of concentration [43], dynamics that stem from intrinsic cultivation factors and hydrophobic fractions [48,49].

Isolated limonene exhibits low activity, shifting the inhibitory protagonism to minor oxygenated compounds [49,50]. In the antifungal scope, the oil inhibited Candida species, resembling the data collected by Lima et al. [51]. Other studies recorded inhibitions at lower concentrations against *Aspergillus niger*, *A. flavus*, *Penicillium* sp., and *Rhizopus* sp., while Gasparetto et al. [52], Oliveira et al. [53], and Menezes Filho et al. [54] attested to the high efficiency of citrus matrices rich in D-limoneno against *A. niger*, *Penicillium sumatrensis*, and *Malassezia furfur*.

The antimicrobial performance of the potato starch films was influenced by both the botanical origin and concentration of the incorporated citrus essential oils. At concentrations ranging from 1 to 2%, films containing lemon essential oil exhibited greater antibacterial activity against the Gram-positive bacterium *S. aureus*, whereas films containing tangerine essential oil were more effective against the fungus *A. niger*. In contrast, none of the formulations inhibited *E. coli*, indicating that the antimicrobial response was both microorganism- and oil-dependent.

Unlike several reports describing a concentration-dependent increase in antimicrobial activity [20,21], the present study did not show a linear response to increasing essential oil concentration, indicating that antimicrobial performance is governed by the availability of bioactive compounds rather than by the total amount of oil incorporated. This behavior suggests that increasing oil concentration may promote droplet coalescence and stronger retention of volatile compounds within the starch matrix, reducing their diffusion and bioavailability [55,56].

Furthermore, the distinct responses observed among lemon, orange, and tangerine essential oils reinforce that citrus oils should not be considered a homogeneous group. Although limonene is the predominant constituent of citrus peel oils, differences in the relative proportions of oxygenated monoterpenes and other minor compounds can influence both antimicrobial potency and release kinetics [57,58]. Similar selective antimicrobial responses have been reported for starch-based films [14] and for gelatin, chitosan, and pectin matrices containing citrus essential oils [11,12,13].

Although the agar diffusion assay is widely employed to evaluate the antimicrobial activity of starch-based films containing essential oils [55,59,60], the hydrophobic nature of these compounds may limit their diffusion through the hydrophilic PDA matrix, potentially underestimating their antifungal activity. Therefore, the inhibition halos observed in this study should be interpreted as an initial screening of antifungal potential. Complementary methods, such as direct-contact or vapor-phase assays, may provide additional information regarding the effectiveness of volatile essential oil components and should be considered in future investigations.

The incorporation of these free oils into the potato starch matrix triggered remarkable physical and optical changes, confirming our working hypothesis that the hydrophobic core rearranges the polysaccharide chain distribution. Film thickness decreased after essential oil incorporation, consistent with the findings by Kumar et al. [23], who reported a reduction in the thickness of corn starch films from 0.1454 to 0.1406 mm after the addition of 2.5% orange essential oil. The authors attributed this behavior to the increased non-aqueous fraction and the formation of a denser structure during solvent evaporation. Similar structural rearrangements were reported by Cai et al. [59], who associated changes in film thickness with the increase in thyme essential oil concentration, reinforcing that the dispersed lipid phase influences polymer chain packing during film formation.

Likewise, transparency decreased significantly compared with the control film, indicating greater light scattering caused by the presence of dispersed oil droplets and increased structural heterogeneity, corroborating previous observations in starch–essential oil systems [59]. Although the overall color difference (ΔE) increased significantly relative to the control, no significant differences were observed among the citrus essential oils, suggesting that botanical origin had little influence on the optical appearance of the films. Similar behavior was reported by Yang et al. [56], who also observed that essential oil incorporation altered film color without significant differences among oil concentrations. Furthermore, the incorporation of hydrophobic compounds reduced film solubility by decreasing the affinity of the starch matrix for water, in agreement with Kumar et al. [23], who attributed this effect to the replacement of hydrophilic interactions by hydrophobic domains within the polymeric network.

The topography and internal morphology elucidated by SEM revealed irregular surfaces and rough cross-sections with dispersed porosities and bubbles at the highest lipid dosages (2%), mimetizing the observations by Basiak, Lenart, and Debeaufort [2] and Song et al. [20]. Such cavities derive from the migration and partial evaporation of the oil during drying [55]. These structural discontinuities weakened the polymeric network, causing reductions in maximum tensile strength and elongation, a behavior analogous to that found by Evangelho et al. [21].

The mechanical behavior of the starch–citrus essential oil films reflects the complex interaction between the dispersed lipid phase and the starch polymeric network. In the present study, the incorporation of tangerine essential oil (1 and 2%, *w*/*w*) significantly reduced tensile strength, whereas lemon essential oil did not significantly affect this parameter, indicating that the mechanical response depends not only on oil concentration but also on its botanical origin and compatibility with the starch matrix. Similar reductions in tensile strength have been reported for corn starch films containing orange essential oil [21], in which the dispersed lipid phase weakened intermolecular cohesion within the polymeric network. In contrast, several studies have described opposite trends.

Cai et al. [59] observed that increasing thyme essential oil concentration enhanced tensile strength from 2.3 to 3.4 MPa, although elongation at break simultaneously decreased from 40.9 to 23.5%, indicating increased matrix rigidity. Likewise, Kumar et al. [23] reported an increase in the maximum tensile load of corn starch films from 329.16 N in the control to 493.21 N after incorporating 2.5% orange essential oil. Song et al. [20] also found simultaneous improvements in water vapor barrier properties, tensile strength (11.18–15.34 MPa), and elongation at break (31.10–36.30%) after incorporating 0.5–2% lemon essential oil into corn starch films.

The XRD diffractogram of the native potato starch (PS) confirmed the characteristic B-type crystalline pattern, exhibiting well-defined diffraction peaks at approximately 5.58°, 15.06°, 17.10°, 19.50°, 22.20°, 24.00°, and 26.10° (2θ), with a crystallinity index (CrI) of 44.89%, which is characteristic of native potato starch granules and agrees well with previous reports for B-type starch [32,61].

After film formation, thermal gelatinization at 80 °C, combined with the presence of glycerol and citric acid, almost completely disrupted the native crystalline organization, reducing the crystallinity index to 2.30% in the control film (PSF). Consequently, the diffraction profiles of the developed films became predominantly amorphous, characterized by broad diffraction halos between approximately 17° and 24° (2θ). Therefore, the diffractograms obtained in this study represent fully gelatinized starch-based films rather than native starch, explaining the marked reduction in the intensity of the characteristic B-type reflections after film formation. Similar structural transitions, involving disruption of the native B-type crystalline organization and the predominance of amorphous domains after gelatinization, have been widely reported for starch-based films [61,62,63,64].

The incorporation of glycerol and citric acid promoted esterification and crosslinking reactions [65], disrupting native hydrogen bonds, reducing the degree of crystallinity, and favoring the rearrangement of the amorphous phase during retrogradation [64]. The incorporation of citrus essential oils further modulated the crystalline organization of the films. At 1% concentration, the essential oils attenuated the diffraction peaks, indicating a reduction in crystalline order, whereas the 2% formulations exhibited increased peak intensity, suggesting greater molecular organization promoted by stronger intermolecular interactions. Tangerine essential oil induced more pronounced reflections at lower concentration, which progressively evolved toward a V-type profile at higher loading, similarly to the structural rearrangements reported for crosslinked starch systems by Wu et al. [66].

FTIR analyses mapped typical bands shared among the constituents: broad hydroxyl (–OH) stretching in acids and polyols at 3500–3200 cm^−1^, aliphatic C–H bonds at 2926 cm^−1^, axial stretching at 2395 cm^−1^, carbonyls (C=O) at 1716 cm^−1^, alkene bonds (C=C) at 1585 cm^−1^ and 1456 cm^−1^, and ester bonds (C–O–C) at 1001 cm^−1^ [22,67,68,69]. These absorption bands are consistent with the characteristic FTIR profile reported for potato starch and gelatinized starch systems, particularly the broad O–H stretching band associated with hydrogen bonding, the C–H stretching vibrations around 2920 cm^−1^, and the polysaccharide fingerprint region between 1200 and 900 cm^−1^ [70]. The overlapping and stability of the bands denote interfacial neutrality and the absence of anomalous chemical interactions, a pattern similar to that documented by Brandelero, Almeida, and Alfaro [69] and Maizura et al. [71]. This inertia preserves the integrity of the active compounds, keeping them unhindered by rigid bonds to exert their fungistatic and bacteriostatic functions.

The thermogravimetric profiles also demonstrate that the incorporation of citrus essential oils did not substantially modify the thermal degradation mechanism of the starch matrix, as all formulations exhibited similar degradation patterns characterized by three distinct mass-loss stages. This behavior indicates that the essential oils were physically incorporated into the polymeric network without promoting significant changes in the thermal decomposition pathway of the gelatinized starch matrix. The first degradation stage is associated with the evaporation of free and bound water due to the hydrophilic nature of starch and glycerol. The second and most pronounced stage, occurring between approximately 280 and 350 °C, corresponds to the depolymerization of starch chains through cleavage of glycosidic linkages and degradation of glycerol, whereas the final stage is related to the decomposition of carbonaceous residues formed during pyrolysis. Similar thermal degradation behavior has been reported for potato starch-based films prepared by the casting method, confirming that these degradation events are characteristic of gelatinized starch matrices plasticized with glycerol [70,72].

The soil burial test confirmed the high biodegradability of the developed potato starch films, with mass losses exceeding 50% after 15 days and near-complete structural disintegration after 45 days. This degradation profile was faster than that reported by Costa et al. [24] for peach palm starch films and comparable to other starch-based biodegradable systems. Chavez-Marquez et al. [73] reported approximately 95% weight loss after 21 days for potato starch films containing molle essential oil and anthocyanins, demonstrating that starch matrices remain highly susceptible to microbial degradation even after the incorporation of bioactive compounds.

Similarly, Pozzebon et al. [74] observed that corn starch films containing lemongrass essential oil maintained their degradability despite the reduction in water solubility promoted by the hydrophobic phase, indicating that the starch matrix remains the primary substrate for soil microorganisms. Unlike Singh et al. [75], who reported that the incorporation of lemongrass oil nanoemulsions delayed the degradation of corn starch nanocomposite films because of the combined antimicrobial activity of the essential oil and the reinforcing effect of montmorillonite nanoclay, the citrus essential oils incorporated into the present potato starch films did not compromise biodegradation.

The biodegradation products also proved to be environmentally safe, as no phytotoxic effects were observed during coriander seed germination. The maintenance of dry biomass and the absence of significant changes in fresh biomass indicate that the degradation products did not impair seed germination or early plant development. Similar findings were reported by Reichert et al. [76], who demonstrated that starch- and cellulose-based biodegradable films do not negatively affect plant growth after soil degradation, reinforcing the environmental suitability of starch-based active packaging materials.

The chemical composition of the citrus essential oils played a decisive role in determining the functional performance of the starch-based bioactive films. Although limonene was the predominant constituent in all three essential oils, their compositional profiles differed markedly. Lemon essential oil exhibited the greatest chemical diversity, containing substantial amounts of β-pinene and γ-terpinene in addition to limonene, whereas orange essential oil was composed almost exclusively of limonene. Tangerine essential oil presented an intermediate profile, combining a high limonene content with moderate proportions of γ-terpinene and other monoterpenes. Previous studies have demonstrated that the antimicrobial efficacy and functional performance of essential oils depend not only on the concentration of the major constituent but also on the synergistic interactions among minor volatile compounds, which can enhance biological activity and modify their interaction with polymeric matrices [77,78,79].

Likewise, the incorporation of chemically distinct essential oils into starch-based films has been reported to influence the physicochemical, mechanical, barrier, and biodegradation properties of biodegradable materials through differences in intermolecular interactions within the polymer network [20,69,80]. Therefore, the results obtained in the present study suggest that the bioactivity, physicochemical characteristics, and biodegradation behavior of the developed films were governed not only by the abundance of limonene but also by the overall chemical complexity of each citrus essential oil, highlighting the importance of considering the complete phytochemical profile when designing starch-based active packaging materials.

From a broader perspective, these findings imply that agri-food waste can be successfully upcycled into high-value functional materials, directly contributing to the circular economy and offering a scalable alternative to fossil-based plastics. Future research directions should investigate the scaling up of the casting method to continuous industrial pilot lines, evaluate the long-term migration kinetics of specific terpenes into real food systems under varied storage temperatures, and explore industrial composting scenarios to fully map the environmental impact lifecycle of these films.

## 5. Conclusions

This study demonstrates the technical and environmental feasibility of developing active films using a potato starch matrix incorporated with citrus peel essential oils (lemon and tangerine). The matrices successfully preserved the functional properties of the bioactive monoterpenes, predominantly D-limonene, conferring relevant antimicrobial action against *Aspergillus niger* and *Staphylococcus aureus*. While the incorporation of the hydrophobic active core induced predictable microstructural rearrangements, reducing tensile strength and increasing the swelling degree, it did not compromise the film-forming capacity, transparency, or water solubility baseline of the starch matrices. The environmental milestones of the developed biomaterials were fully validated, achieving rapid degradation in soil with more than 50% mass loss after 15 days and near-complete disintegration within 45 days. The degradation by-products demonstrated zero ecotoxicological risk, allowing for normal germination and physical development of coriander plants. Therefore, these active starch-based films present a viable, circular economy alternative to fossil-based materials in the food packaging industry, successfully balancing functional preservation with an eco-friendly end-of-life cycle. Future research directions should focus on scaling up the casting process to continuous industrial pilot lines, establishing the migration kinetics of specific volatile compounds into various food simulants under distinct storage temperatures, and evaluating the long-term shelf-life extension efficiency of these films when applied to real perishable food products.

## Figures and Tables

**Table 1:** Physical properties and extraction yields of citrus essential oils obtained by hydrodistillation.

Essential Oil Source	Botanical Name	Initial Biomass (g)	Extracted Volume (mL) ^1^	Extraction Yield (%, *v*/*w*) ^1^	Density (g/cm^3^) ^1^
Lemon	*Citrus latifolia* Tanaka	300.0	2.53 ± 0.06 ^b^	0.84 ± 0.02 ^b^	0.842 ± 0.001 ^b^
Orange	*Citrus sinensis*	300.0	5.40 ± 0.10 ^a^	1.80 ± 0.03 ^a^	0.839 ± 0.001 ^c^
Tangerine	*Citrus reticulata*	300.0	2.10 ± 0.06 ^c^	0.70 ± 0.02 ^c^	0.847 ± 0.001 ^a^

^1^ Values represent means ± standard deviation (n = 3). Different superscript letters within the same column indicate statistically significant differences (*p* < 0.05) determined by Tukey’s post hoc test.

**Table 2:** Comparative volatile chemical composition (% of relative area) of lemon (*Citrus latifolia*), orange (*Citrus sinensis*), and tangerine (*Citrus reticulata*) essential oils determined by GC-MS.

No.	Retention Time (min) ^1^	Active Compound	Chemical Class	Lemon (%) ^2^	Orange (%) ^2^	Tangerine (%) ^2^
1	4.37–4.82	α-Thujene	Monoterpene Hydrocarbon	1.38	—	0.66
2	4.45–4.95	α-Pinene	Monoterpene Hydrocarbon	3.91	0.94	2.84
3	5.32–6.01	β-Pinene	Monoterpene Hydrocarbon	15.96	0.27	0.88
4	5.40–5.93	Sabinene	Monoterpene Hydrocarbon	2.40	—	1.72
5	5.79–6.45	β-Myrcene	Monoterpene Hydrocarbon	1.86	2.64	1.60
6	7.58	Octanal	Aliphatic Aldehyde	—	—	1.28
7	7.10–7.91	Limonene	Monoterpene Hydrocarbon	57.79	95.78	76.22
8	8.06–8.95	γ-Terpinene	Monoterpene Hydrocarbon	16.00	—	9.83
9	9.31	o-Cymene	Monoterpene Hydrocarbon	—	—	4.14
10	10.05–11.23	Linalool	Oxygenated Monoterpene	0.69	0.37	0.83
Total				100.00	100.00	100.00

^1^ Retention times represent the elution range observed across the analyzed citrus samples. ^2^ Normalized values calculated by considering exclusively the identified natural active phytocompounds (and excluding any non-natural synthetic artifacts) to sum exactly 100.00% across all samples.

**Table 3:** Antibacterial activity (zone of inhibition) and minimum inhibitory concentration (MIC) of citrus peel essential oils.

Essential Oil Source	*Escherichia coli*		*Staphylococcus aureus*	
	Zone of Inhibition (mm) ^1^	MIC (%, *v*/*v*)	Zone of Inhibition (mm) ^1^	MIC (%, *v*/*v*)
Lemon	13.23 ± 0.15 ^a^	16.0	13.25 ± 0.12 ^a^	16.0
Orange	11.12 ± 0.08 ^c^	ND ^2^	ND ^2^	ND ^2^
Tangerine	12.18 ± 0.10 ^b^	ND ^2^	12.31 ± 0.11 ^b^	ND ^2^

^1^ Values represent means ± standard deviation (n = 3). Different superscript letters within the same column indicate statistically significant differences (*p* < 0.05) determined by Tukey’s post hoc test. ^2^ ND: Not Detected (no visible inhibition zone).

**Table 4:** Antifungal activity (zones of inhibition) and minimum inhibitory concentration (MIC) of citrus peel essential oils and positive control.

Antifungal Agent	*Aspergillus niger* (mm) ^1^		*Bread fungi* (mm) ^1,4^		MIC (%/mg·mL^−1^) ^2^
Lemon (*Citrus latifolia Tanaka*)	0.00 ± 0.00 ^d^	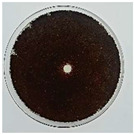	16.59 ± 0.00 ^c^	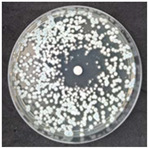	2% (20.2 mg·mL^−1^)
Orange (*Citrus sinensis*)	22.44 ± 2.42 ^c^	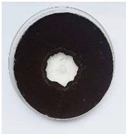	23.41 ± 0.00 ^b^	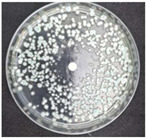	2% (20.0 mg·mL^−1^)
Tangerine (*Citrus reticulata*)	27.32 ± 3.01 ^b^	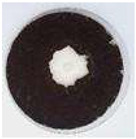	ND ^3^	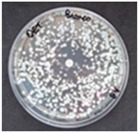	4% (33.8 mg·mL^−1^)
Ketoconazole (20.0 mg·mL^−1^)	45.35 ± 4.91 ^a^	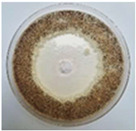	-	-	-

^1^ Values represent means ± standard deviation (n = 3). Different superscript letters within the same column indicate statistically significant differences (*p* < 0.05) determined by Tukey’s post hoc test. ^2^ MIC values are expressed as percentage volume/volume (%, *v*/*v*) with corresponding mass concentrations indicated in parentheses, calculated using specific oil densities. ^3^ ND: Not Detected (no visible inhibition zone). ^4^ Visual assessment indicated fungistatic activity (formation of more than 3 CFUs).

**Table 5:** Antimicrobial response of potato starch films incorporated with citrus peel essential oils.

Film Formulation ^1^	Bacteria		Fungus
	*S. aureus* (Gram Positive)	*E. coli* (Gram Negative)	*A. niger*
PSF	−	−	−
PSF-LEO 0.5%	+	−	−
PSF-LEO 1.0%	+	−	−
PSF-LEO 2.0%	−	−	+
PSF-OEO 0.5%	−	−	−
PSF-OEO 1.0%	−	−	−
PSF-OEO 2.0%	−	−	−
PSF-TEO 0.5%	−	−	−
PSF-TEO 1.0%	−	−	+
PSF-TEO 2.0%	−	−	+

^1^ PSF: pure potato starch film; LEO: lemon essential oil; OEO: orange essential oil; TEO: tangerine essential oil. (+) indicates a visible zone of inhibition partial of microbial growth around the film matrix; (−) indicates normal microbial proliferation with no inhibitory response.

**Table 6:** Physical and optical parameters of potato starch films incorporated with citrus essential oils and commercial control.

Films	Thickness (mm)	L*	a*	b*	∆E
CF	0.01 ± 0.00 ^g^	31.23 ± 0.09 ^h^	−2.42 ± 0.17 ^a^	−0.75 ± 0.12 ^c^	2.06 ± 0.23 ^d^
PSF	0.15 ± 0.03 ^b,c^	61.24 ± 0.00 ^d^	−2.32 ± 0.02 ^a^	0.54 ± 0.00 ^a^	5.69 ± 0.30 ^a^
PSF-LEO 0.5%	0.13 ± 0.04 ^d^	58.47 ± 0.12 ^b^	−2.41 ± 0.22 ^a^	−0.39 ± 0.22 ^b^	3.36 ± 0.18 ^b,c^
PSF-LEO 1%	0.14 ± 0.02 ^c,d^	60.02 ± 0.08 ^c^	−2.73 ± 0.14 ^b^	−0.89 ± 0.16 ^c^	3.78 ± 0.18 ^b,c^
PSF-LEO 2%	0.15 ± 0.02 ^f^	58.66 ± 0.11 ^b^	−2.91 ± 0.09 ^e^	−0.53 ± 0.16 ^b,c^	3.24 ± 0.29 ^c^
PSF-OEO 0.5%	0.12 ± 0.01 ^d,e^	59.73 ± 0.08 ^b^	−2.80 ± 0.18 ^d^	−0.87 ± 0.12 ^c^	3.59 ± 0.39 ^b,c^
PSF-OEO 1%	0.10 ± 0.01 ^b^	60.40 ± 0.13 ^c^	−2.65 ± 0.16 ^a^	−1.99 ± 0.20 ^d^	3.51 ± 0.29 ^b,c^
PSF-OEO 2%	0.11 ± 0.03 ^e,f^	58.59 ± 0.06 ^b^	−2.77± 0.08 ^c^	−2.85 ± 0.13 ^e^	1.51 ± 0.39 ^d^
PSF-TEO 0.5%	0.13 ± 0.03 ^d,e^	59.06 ± 0.04 ^b^	−2.81 ± 0.25 ^e^	−0.33 ± 0.19 ^b^	3.62 ± 0.39 ^b,c^
PSF-TEO 1%	0.17 ± 0.04 ^f^	58.98 ± 0.16 ^b^	−2.82 ± 0.33 ^a^	−0.19 ± 0.06 ^b^	3.68 ± 0.29 ^b,c^
PSF-TEO 2%	0.21 ± 0.03 ^a^	56.42 ± 0.12 ^d^	−2.36 ± 0.28 ^a^	0.37 ± 0.27 ^a^	3.98 ± 0.28 ^b^

Legend: ∆E, total color difference. Different superscript letters within the same column indicate statistically significant differences (*p* < 0.05) determined by Tukey’s post hoc test.

**Table 7:** Water content, water solubility, and degree of swelling of potato starch-based films incorporated with citrus peel essential oils.

Films	WC (%)	SD (%)	WS (%)
PSF	17.17 ± 0.87 ^a^	55.19 ± 5.01 ^b^	31.11 ± 1.92 ^a^
PSF-LEO 0.5%	19.16 ± 5.42 ^a^	74.07 ± 6.42 ^a^^b^	33.33 ± 0.00 ^a^
PSF-LEO 1%	24.44 ± 7.70 ^a^	100.00 ± 43.30 ^a^^b^	33.33 ± 14.43 ^a^
PSF-LEO 2%	23.33 ± 8.82 ^a^	111.11 ± 19.25 ^a^	31.39 ± 12.81 ^a^
PSF-OEO 0.5%	12.86 ± 2.47 ^a^	66.67 ± 0.00 ^a^^b^	33.33 ± 0.00 ^a^
PSF-OEO 1%	22.86 ± 4.95 ^a^	85.00 ± 13.23 ^a^^b^	23.33 ± 2.89 ^a^
PSF-OEO 2%	23.33 ± 2.89 ^a^	100.00 ± 0.00 ^a^^b^	30.56 ± 4.81 ^a^
PSF-TEO 0.5%	17.78 ± 1.92 ^a^	71.67 ± 10.41 ^a^^b^	28.33 ± 10.41 ^a^
PSF-TEO 1%	23.41 ± 6.11 ^a^	112.22 ± 10.72 ^a^	37.78 ± 20.37 ^a^
PSF-TEO 2%	22.41 ± 2.51 ^a^	99.07 ± 23.62 ^a^^b^	33.13 ± 4.47 ^a^

Legend: WC: water content; WS: water solubility; SD: degree of swelling. Values within columns followed by the same superscript letter do not differ significantly (*p* > 0.05) according to Tukey’s post hoc test.

**Table 8:** Quantitative mass-loss percentage (%) of commercial cellophane film (CF) and active potato starch-based films during the 45-day soil burial experiment.

Films	Day 5	Day 10	Day 15	Day 30	Day 45	Mean
CF	0.0 ± 0.0	0.0 ± 0.0	0.0 ± 0.0	0.0 ± 0.0	0.0 ± 0.0	0.0 ^c^
PSF	35.4 ± 6.01	40.1 ± 0.68	39.1 ± 27.5	53.3 ± 6.3	35.9 ± 9.4	40.76 ^b^
PSF-LEO 1%	40.1 ± 2.40	43.7 ± 0.8	66.7 ± 15.7	69.1 ± 17.2	91.9 ± 7.3	62.3 ^a^
PSF-LEO 2%	48.1 ± 3.2	37.5 ± 22.4	79.2 ± 7.2	42.3 ± 17.5	92.6 ± 12.8	59.94 ^a^
PSF-TEO 1%	44.8 ± 5.0	44.8 ± 4.5	48.7 ± 4.5	56.4 ± 19.2	53.7 ± 3.6	49.68 ^ab^
PSF-TEO 2%	45.7 ± 4.2	51.3± 6.5	74.7 ± 21.8	69.0 ± 11.2	66.4 ± 10.7	61.42 ^a^
Mean	35.68 ^a^	36.65 ^a^	51.40 ^a^	48.34 ^a^	56.74 ^a^	

Note: Values within columns followed by the same superscript letter do not differ significantly (*p* > 0.05) according to two-way ANOVA followed by Tukey’s post hoc test.

**Table 9:** Biomass allocation parameters of coriander seedlings (*Coriandrum sativum*) developed in soil contact with commercial control and active starch-based films after 20 days.

Sample	Fresh Mass (g)	Dry Mass (g)
CF	0.07 ± 0.01	0.01 ± 0.00
PSF	0.06 ± 0.01	0.01 ± 0.00
PSF–LEO 1%	0.05 ± 0.00	0.01 ± 0.00
PSF–LEO 2%	0.06 ± 0.01	0.01 ± 0.00
PSF–TEO 1%	0.05 ± 0.00	0.01 ± 0.00
PSF–TEO 2%	0.04 ± 0.00	0.01 ± 0.00

## Data Availability

The data presented in this study are available upon request from the corresponding author.

## Supplementary Materials

The following supporting information can be downloaded at: https://www.mdpi.com/article/10.3390/polym18141794/s1, Table S1: Antifungal activity and minimum fungicidal concentration of lemon (*Citrus latifolia* Tanaka) peel essential oil; Table S2: Antifungal activity and minimum fungicidal concentration of orange (*Citrus sinensis*) peel essential oil; Table S3: Antifungal activity and minimum fungicidal concentration of tangerine (*Citrus reticulata*) peel essential oil; Table S4: Antibacterial activity of potato starch films enriched with citrus peel essential oil against *S. aureus*; Table S5: Antibacterial activity of potato starch films incorporated with citrus peel essential oil against *Escherichia coli*; Table S6: Antifungal activity of potato starch films incorporated with citrus peel essential oil against *Aspergillus niger*.

## Abbreviations

CF	Commercial cellophane film
GRAS	Generally Recognized as Safe
FDA	Food and Drug Administration
LEO	Lemon essential oil
OEO	Orange essential oil
PSF	Potato starch film
TEO	Tangerine essential oil
