# Supplementary material for: Active Starch Films Incorporated with Citrus Essential Oils: Properties, Bioactivity, and Biodegradability
Source: Polymers (Basel). 2026 Jul 22;18(14):1794. doi: 10.3390/polym18141794 (PMC13417027; doi:10.3390/polym18141794)
Supplement: Supplementary file 1 [file polymers-18-01794-s001.zip › polymers-4389217-supplementary.pdf]

Material supplementary

Table S1. Antifungal activity and minimum fungicidal concentration of lemon (*Citrus latifolia* Tanaka) peel essential oil.

**Lemon essential oil (%)**	**Concentration (mg/mL)**	CFU/ **mL**		**Antifungal activity**
8	81.0	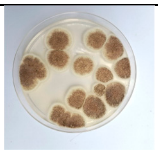	13	Fungistatic
4	40.5	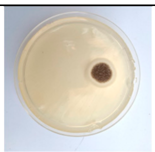	1	Fungicidal
2	20.2	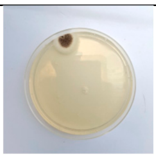	1	MFC[1]
1	10.1	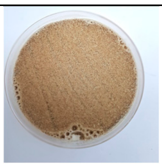	UNC	Resistant
0.5	5.1	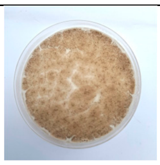	UNC	Resistant
0.25	2.5	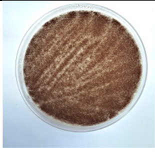	UNC	Resistant
0.0125	1.2	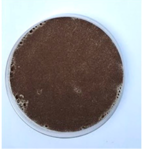	UNC	Resistant

Legend: MFC: [1]Minimum Fungicidal Concentration (no fungal growth or formation of fewer than 3 CFUs); Fungistatic (formation of more than 3 CFUs); UNC: Uncountable; Resistant (normal fungal growth).

Table S2. Antifungal activity and minimum fungicidal concentration of orange (*Citrus sinensis*) peel essential oil.

**Orange essential oil (%)**	**Concentration (mg/mL)**	CFU/ **mL**		**Antifungal activity**
8	80.0	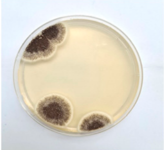	4	Fungistatic
4	40.0	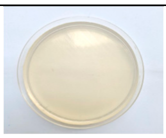	0	Fungicidal
2	20.0	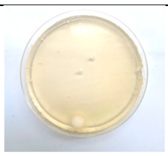	0	MFC[1]
1	10.0	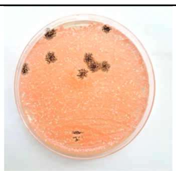	7	Fungistatic
0.5	5.0	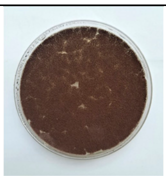	UNC	Resistant
0.25	2.5	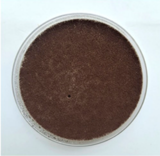	UNC	Resistant
0.0125	1.2	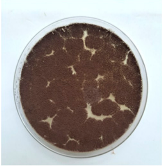	UNC	Resistant

Legend: [1]MFC: Minimum Fungicidal Concentration (no fungal growth or formation of fewer than 3 CFUs); Fungistatic (formation of more than 3 CFUs); UNC: Uncountable; Resistant (normal fungal growth).

Table S3. Antifungal activity and minimum fungicidal concentration of orange (*Citrus reticulata)* peel essential oil.

**Tangerine essential oil (%)**	**Concentration (mg/mL)**	CFU/ **mL**		**Antifungal activity**
8	116.0	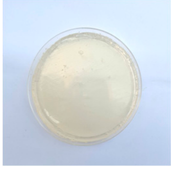	0	Fungicidal
4	58.0	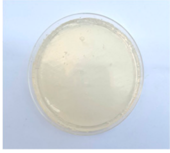	0	MFC[1]
2	29.0	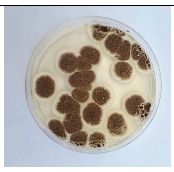	20	Fungistática
1	14.5	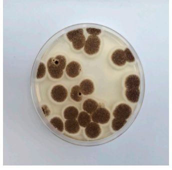	24	Fungistatic
0.5	7.2	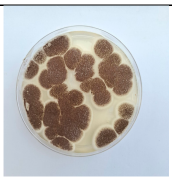	34	Fungistatic
0.25	3.6	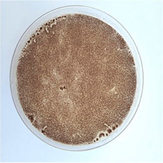	UNC	Resistant
0.0125	1.8	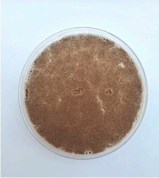	UNC	Resistant

Legend: MFC: Minimum Fungicidal Concentration (no fungal growth or formation of fewer than 3 CFUs); Fungistatic (formation of more than 3 CFUs); UNC: Uncountable; Resistant (normal fungal growth).

Table S4-Antibacterial activity of potato starch films enriched with citrus peel essential oil against *S. aureus.*

Films	Bacterial activity	
PSF	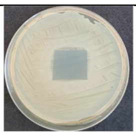	-
PSF – LEO 0.5%	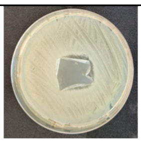	+
PSF – LEO 1.0%	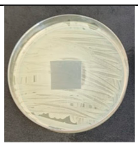	+
PSF – LEO 2.0%	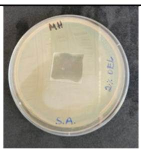	-
PSF – OEO 0.5%	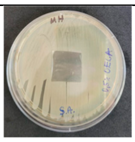	-
PSF – OEO 1.0%	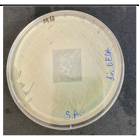	-
PSF – OEO 2.0%	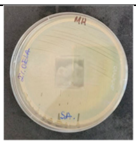	-
PSF – TEO 0.5%	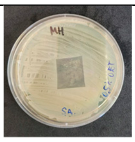	-
PSF – TEO 1.0%	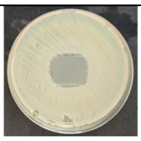	+
PSF – TEO 2.0%	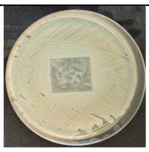	-

Table S5- Antibacterial activity of potato starch films incorporated with citrus peel essential oil against *Escherichia coli.*

Films	Bacterial activity	
PSF	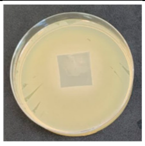	-
PSF – LEO 0.5%	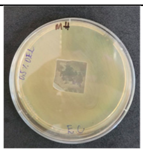	-
PSF – LEO 1.0%	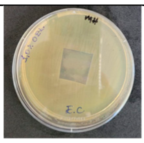	-
PSF – LEO 2.0%	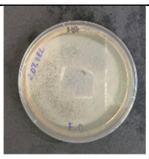	-
PSF – OEO 0.5%	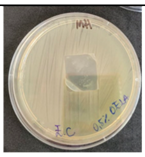	-
PSF – OEO 1.0%	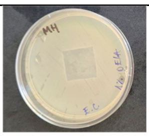	-
PSF – OEO 2.0%	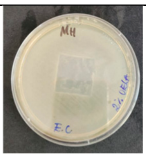	-
PSF – TEO 0.5%	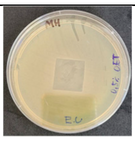	-
PSF – TEO 1.0%	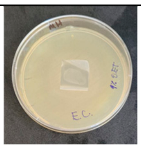	-
PSF – TEO 2.0%	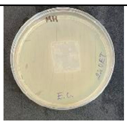	-

Table S6- Antifungal activity of potato starch films incorporated with citrus peel essential oil against *Aspergillus niger*.

Films	Bacterial activity	
PSF	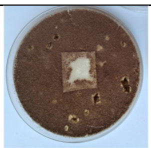	INC
PSF – LEO 0.5%	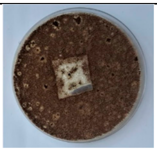	INC
PSF – LEO 1.0%	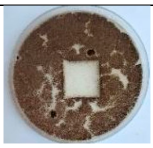	INC
PSF – LEO 2.0%	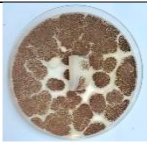	28
PSF – OEO 0.5%	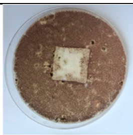	INC
PSF – OEO 1.0%	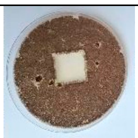	INC
PSF – OEO 2.0%	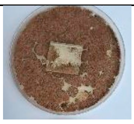	INC
PSF – TEO 0.5%	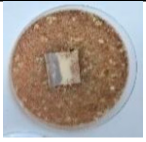	INC
PSF – TEO 1.0%	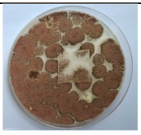	27
PSF – TEO 2.0%	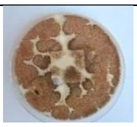	20
